# Optimization of Landscape Services under Uncoordinated Management by Multiple Landowners

**DOI:** 10.1371/journal.pone.0086001

**Published:** 2014-01-17

**Authors:** Miguel Porto, Otília Correia, Pedro Beja

**Affiliations:** 1 Centro de Biologia Ambiental, Departamento de Biologia Vegetal, Faculdade de Ciências, Universidade de Lisboa, Lisboa, Portugal; 2 EDP Biodiversity Chair, CIBIO - Research Center in Biodiversity and Genetic Resources/InBIO, University of Porto, Vairão, Portugal; 3 CIBIO - Research Center in Biodiversity and Genetic Resources/InBIO, University of Porto, Vairão, Portugal; Indiana University, United States of America

## Abstract

Landscapes are often patchworks of private properties, where composition and configuration patterns result from cumulative effects of the actions of multiple landowners. Securing the delivery of services in such multi-ownership landscapes is challenging, because it is difficult to assure tight compliance to spatially explicit management rules at the level of individual properties, which may hinder the conservation of critical landscape features. To deal with these constraints, a multi-objective simulation-optimization procedure was developed to select non-spatial management regimes that best meet landscape-level objectives, while accounting for uncoordinated and uncertain response of individual landowners to management rules. Optimization approximates the non-dominated Pareto frontier, combining a multi-objective genetic algorithm and a simulator that forecasts trends in landscape pattern as a function of management rules implemented annually by individual landowners. The procedure was demonstrated with a case study for the optimum scheduling of fuel treatments in cork oak forest landscapes, involving six objectives related to reducing management costs (1), reducing fire risk (3), and protecting biodiversity associated with mid- and late-successional understories (2). There was a trade-off between cost, fire risk and biodiversity objectives, that could be minimized by selecting management regimes involving ca. 60% of landowners clearing the understory at short intervals (around 5 years), and the remaining managing at long intervals (ca. 75 years) or not managing. The optimal management regimes produces a mosaic landscape dominated by stands with herbaceous and low shrub understories, but also with a satisfactory representation of old understories, that was favorable in terms of both fire risk and biodiversity. The simulation-optimization procedure presented can be extended to incorporate a wide range of landscape dynamic processes, management rules and quantifiable objectives. It may thus be adapted to other socio-ecological systems, particularly where specific patterns of landscape heterogeneity are to be maintained despite imperfect management by multiple landowners.

## Introduction

In his seminal paper, Hardin [1] hypothesized that a system based on a common resource that is not managed will inevitably tend to exhaustion as a consequence of every user maximizing its own benefit. A landscape used by multiple landowners can be viewed as such a common resource [2], where the resource units may be equated to the products and services the landscape provides [3], [4]. Many of these products and services, including those of direct interest to the landowners such as fire risk regulation [5]–[7] and water quality regulation [8], are dependent on whole-landscape structure [4], [9]. However, landowners maximizing their own benefit at the scale of individual private properties may fail to maintain key structural features at the landscape scale, thus compromising the delivery of valuable ecosystem services [4], [10].

Achieving favorable patterns of landscape composition and configuration is often regarded as a problem of optimizing the spatial distribution and temporal scheduling of management activities, so as to fulfill a given set of objectives [11]–[15]. For instance, optimization of forest landscape management often involves the development of management schedules maximizing sustainable yields, maintaining successional heterogeneity or increasing fire resistance [16]–[18]. Although this approach is feasible where landscapes are closely managed by a single or very few institutions, either public or private, it may be impossible to implement in multi-ownership landscapes, i.e. patchworks of private properties with different owners, where there is little direct control over each landowner's management decisions [19]. Avoiding a landscape level “tragedy of the commons” [1] may thus require landowners to follow a set of prescribed rules, which may be implemented through governmental land use restrictions and management regulations [20], voluntary incentive schemes [10], [21]–[24], or even self-regulation [25]. Whatever the implementation mechanism, however, a key challenge is to optimize rules that once implemented by landowners will contribute to successfully achieving landscape scale objectives that are relevant at the individual and societal levels [19], [26].

Designing management rules for multi-ownership landscapes is particularly difficult, mainly because individual landowners may vary widely in their response to a common set of rules [27], [28], and this variation cannot be controlled or accurately predicted [23], [27]. The inherent unpredictability of the system thus precludes the use of most methods designed to optimize landscape management, which assume that rules will be strictly implemented according to a fixed plan (e.g. [7], [12], [29]). In reality, however, individual landowners may decide to ignore the rules altogether due to poor enforcement, or they may vary in the rigor of their implementation due to individual preferences or economic constraints [23], [30], [31]. As a consequence, the spatial and temporal scheduling of management activities designed through conventional optimization approaches would be only loosely implemented by individual landowners, which may result in landscapes widely different from those initially foreseen. Furthermore, these landscapes would be dynamic due to the spatial and temporal variations in landowners' decisions and natural processes, making it unlikely that an optimal landscape composition and configuration can realistically be achieved, in marked contrast to the solutions normally reached by landscape optimization methods. Solving these problems requires optimization approaches that explicitly integrate the stochasticity inherent to landowners' decisions, and the concurrent dynamics in landscape structure, in the process of designing solutions [32], [33].

This paper describes an approach for optimizing management rules under the constraint of stochastic landowners' decisions in a dynamic landscape, illustrating its application to a problem of Mediterranean forest landscape management for increasing fire resistance and promoting biodiversity conservation. The problem was based on a well-studied upland cork oak, *Quercus suber*, landscape in southern Portugal [34]–[39], where cork is the main forest product and mechanical clearing of understory vegetation to reduce fire risk is the main management action. Management is undertaken at the scale of individual properties depending on landowner decisions, usually occurring at 9-year intervals in association with the cork extraction cycle, though in many stands it may either be absent or very sporadic. Fuel management is thus rather inefficient in this fine-grained (<10 ha) multi-ownership landscape, because it is conducted at the scale of individual properties, with little or no coordination among neighbors, and there is accumulation of fuel materials in properties that are unmanaged for long periods. On the other hand, although unmanaged stands increase fire risk, they are critical for the conservation of biodiversity, which benefits from complex mosaics of forest stands with understory vegetation in different successional stages [35]–[38]. Consideration of biodiversity conservation is mandatory in this landscape, because it is included in a Site of Community Importance classified under the European Directive 92/43/EEC. The problem is thus to design a management regime of understory vegetation clearing that increases landscape resistance to fire propagation, while maintaining the heterogeneous mosaic required for biodiversity conservation. Although the optimization approach presented here was designed to solve a particular problem, it may be applicable to other multi-ownership forest landscapes, as well as other socio-ecological systems where the conservation of common landscape resources is influenced by the uncoordinated decisions of multiple landowners.

## Methods

### The approach

The problem addressed in this study involves the design of simple management rules that during N years of implementation by individual landowners in a multi-ownership landscape, cumulatively result in the best compromise in achieving multiple and conflicting objectives simultaneously. Variation in the responses of individual landowners to management rules causes uncertainties in the spatial pattern and temporal dynamics of landscape composition and configuration, thus making the problem inherently stochastic. To explicitly integrate the stochasticity in landowners' decisions, the study used a simulation-optimization approach [11], [14], [40] where the objective function of an optimization algorithm integrates a landscape simulator and analyzer. Simulation-optimization finds the best configurations of decision variables for a given system, where the performance is evaluated based on the output of a computer simulation model of the system [11]. The optimization is achieved through an iterative process, which involves (a) choosing a solution, which is then (b) evaluated through simulation as to its performance in fulfilling the given objectives, and then (c) passed back to the optimization procedure which uses the simulation results to assign a measure of fitness to the solution [11]. The details of the latter step depend on the optimization algorithm that is used.

The multi-objective optimization approach used in this study was based on the concept of Pareto optimality [41], thus explicitly recognizing that there is no single solution for problems requiring the simultaneous optimization of multiple objectives (e.g. [12], [42]). This was considered more adequate than the conventional weighing of objectives and their subsequent conversion into a scalar-valued function that is optimized (e.g. [6], [7], [18]), because objectives often conflict and because weighing the relative importance of different objectives involves a large degree of subjectivity and is generally difficult to justify [12], [43], being a decision that should be left to the land manager rather than be taken by the investigator.

The concept of Pareto optimality is based on finding the Pareto frontier, which is the set of non-dominated solutions to the problem [44]. A solution is defined to be non-dominated if there exists no other feasible solution that will give an improvement in one objective without a subsequent degradation in at least one other objective [45]. The method thus provides a range of multiple alternatives before the relative importance of the objectives is specified, which can then be used by decision makers to set preferences and re-examining both them and the proposed management model (e.g. [12], [46]).

Calculating the entire non-dominated Pareto frontier by systematically evaluating the entire feasible solution space is computationally prohibitive due to the very large number of combinations of values for the decision variables. Therefore, a genetic algorithm was used as a search process to converge to an approximation of the non-dominated Pareto frontier (e.g. [12], [47], [48]). This is achieved through simultaneous optimization of a vector-valued objective function in order to find the groups of decision variable values (the optimal set) that optimize the management objectives [12]. Genetic algorithms search for the best solutions to a given problem by applying evolutionary principles to a population of candidate solutions over N generations, giving “competitive advantage” to those solutions that best fulfill the objectives as a whole [45]. Each solution is described by a set of decision variables, which are progressively optimized throughout the process. The core of a genetic algorithm is its objective function, which calculates the values of the objectives based on the variables that are being optimized.

### Definition of objectives

The landscape level objectives considered in this study for upland cork oak forests of southern Portugal were (1) fire risk minimization, (2) maximization of biodiversity value, and (3) minimization of management costs. Fire risk was estimated from three surrogate variables related to the amount and spatial distribution of forest fuels across the landscape, which are known to influence fire initiation, propagation and severity. It was thus assumed that minimization of fire risk over a given period implies (1.1) minimization of the maximum annual fuel load across the landscape; (1.2) maximization of the minimum annual edge contrast between areas with different fuel loads, i.e. increase fuel discontinuities [49] and compartmentation [5]; and (1.3) maximization of the minimum annual fuel concentration in a few small areas [5], [6]. These three surrogate variables were summarized along the given period with their overall minimum/maximum values instead of the mean in order to avoid years of extreme fire risk, which would not be captured with a mean. Total fuel load (1.1) was quantified simply by computing the amount of understory biomass of fine materials and leaves (kg/ha) across the landscape, ignoring tree biomass because this was not affected by fuel management. Edge contrast (1.2) and concentration (1.3) of fuel loads across the landscape were estimated using continuous surface metrics [50], which are extensions of the classical patch-based metrics that do not require discretization into patches whose boundaries are often arbitrary [6]. Edge contrast (1.2) was quantified using the variance of edge contrast metric (“root mean square slope”; [50]), which was computed as the variance of the absolute differences in understory biomass between all pairs of adjacent 1-ha cells, showing the highest values when there is strong spatial heterogeneity in biomass distribution (i.e. spatial fragmentation). Fuel concentration (1.3) was estimated using the surface skewness metric [50], which was quantified as the skewness coefficient of understory biomass across all 1-ha cells. This is a non-spatial measure of asymmetry about the mean, showing the highest values when the majority of cells have low biomass and just a few have high biomass. These surrogate variables were used instead of fire spread simulations due to the computational burden of coupling stochastic landscape dynamics with stochastic fire spread simulations [12].

Biodiversity impacts were estimated from two surrogate variables quantifying the area occupied by cork oak stands with mid- (30–60 years) and late-successional (>60 years) understories. These variables were based on the observation that a range of plant and animal species are associated with complex and multi-layered understories, which take a long time to recover after management [35]–[38]. Therefore, there is a risk that widespread and recurrent fuel management across the landscape might eliminate habitat conditions for these species, with negative consequences for biodiversity. A variable reflecting habitat availability for early-successional and edge species was not considered, because it was assumed that the objective of maximizing habitat for these species was equivalent to minimizing fire risk, as both involve the clearing of understory vegetation. It was thus assumed that minimization of biodiversity impacts implies the maximization of the minimum area attained by stands with mid- and late-successional understories across the simulation period.

Minimizing the cost of management was also included as an objective, because an optimal solution for increasing landscape resistance to fire risk would be to manage the entire landscape, but this is not feasible in practice, besides having strongly negative ecological consequences. The total area managed each year was used as a surrogate of management cost, assuming that costs are constant across the landscape. Although this is a simplification, it was considered reasonable because the optimization exercise was based on simulated landscapes that were homogeneous in all respects except understory age. Maximizing economic return was not considered as an objective because this parameter does not depend on the type of management undertaken.

### Definition of optimization decision variables

The interval in years that a given landowner should leave between two consecutive operations of understory clearing was the basic decision variable used in optimization. This variable was selected because it can be easily prescribed and then controlled by an external or internal authority, it reflects the main management decision taken by landowners in the study area, and it has major direct consequences for the landscape level objectives. Management intervals were constrained to vary between 3 and 100 years, with the lower limit corresponding to a value rarely observed in the study area, and the upper limit corresponding to absence of management during the simulation period. Although an adaptive management approach could possibly provide better outcomes, our goal was to assess to what extent reasonable outcomes can be achieved in a system where resources are insufficient to monitor the effectiveness of management guidelines.

Based on the management interval, five strategies of increasing organizational complexity were defined, in order to explore the trade-off between strategy complexity and performance in achieving objectives: i) a single group strategy, where all landowners are asked to manage with a given interval; and multiple group strategies, where landowners are divided in (ii) two, (iii) three, (iv) four, and (v) five groups according to given proportions, each of which is asked to manage with a different given interval, but it is not defined who belongs to each group. The decision variables hence included the management interval to be implemented by each of the five groups of landowners, and the proportions of landowners in each management group. The number of management groups was not explicitly optimized, but it emerges naturally in the genetic algorithm when all landowners are concentrated in a reduced subset of groups (i.e., four or less). Division in groups was used because observations from the study area suggest that groups of landowners manage their land differently, probably depending on personal interests and social and economic constraints. For instance, many landowners live in coastal towns far from their property, and so they are likely to undertake management operations less frequently than landowners living in small parishes close to their land. Hence, in practical terms, a more complex strategy may, in some cases, be more likely to be implemented correctly. If landowner groups (in respect to management interval) naturally exist in a given real case study, designing group-specific management guidelines that are not spatially explicit allows landowners to self-organize in groups in a way that the overall effort is minimized. Hence, a multiple group strategy in which it is irrelevant who manages with a given interval as long as the overall proportion is somewhat controlled, may possess advantages in some cases, being worth exploring to what extent it helps in achieving the landscape level objectives.

### Landscape simulation

The study focused on randomly generated landscapes that were based on the ecological and socio-economic characteristics of cork oak forest landscapes in Serra do Caldeirão, Southern Portugal (for details see [35]–[38]). Landscapes were an 18×18 km square of 1-ha hexagonal cells, which was randomly partitioned by different landowners into landholdings, with areas approximately following a Gamma distribution with mean = 20 and variance = 100. Gamma distribution was used because there is no *a priori* knowledge about the expected distribution of the landholding area except that it is strictly positive. The algorithm for landscape partitioning into landholdings involved randomly selecting a seed cell for an owner and expanding it randomly to contiguous cells, until the area of the patch reached a random value taken from the Gamma distribution, or until there were no more unassigned contiguous cells left. This process was repeated until all cells had an owner assigned. A pool of 2000 random landscapes was previously generated to avoid the overhead burden of generating a new landscape in each solution evaluation. To simulate the heterogeneous vegetation mosaic characteristic of the study area, each landholding was assigned an age since the last understory clearing event, which was sampled from a uniform distribution between 0 and 70 years. Each landholding was thus considered a management unit, assuming that every landowner will manage his entire property at once. Simulated landscapes were used instead of a real landscape, due to practical difficulties in assessing the boundaries of individual properties and to lack of data on the management history of every property. Furthermore, the use of simplified simulated landscapes was expected to enhance the generality of the results and thus its application to other socio-ecological systems.

Landscape dynamics were simulated by incorporating the disturbance-succession processes associated with fuel management by individual landowners. According to each management strategy, each landowner cleared the understory vegetation at specified time intervals, independently of other landowners. For a given landholding, vegetation clearing started either at time zero if the understory was older than the prescribed management time interval at the simulation outset, or when it reached the age to be cleared if it was younger than the prescribed management interval. Uncertainty in landowner responses to management rules was introduced by specifying the management interval of each landowner as a random variable instead of a fixed value, following a Gamma distribution with mean equal to the specified management rule and variance computed such that the shape parameter was kept constant and equal to 100 (Figure S1). The purpose was to make uncertainty larger at larger intervals (i.e. the variance positively correlated with the mean).

It was assumed that mechanical fuel management removes all understory woody vegetation except cork oaks, and that after management the vegetation recovers following the successional pathway described by [36], with biomass accumulation following the curve described by [39]. Although this curve was based on data for the first 70 years after understory clearing, we deemed it reasonable to extrapolate up to 100 years, because variation after about the first 50 years was very slight. Assumptions regarding the biomass accumulation curve could also be considered simplistic due to spatial and temporal heterogeneities in vegetation successional pathways [51], [52]. However, it was expected that they still provide a useful approximation to the landscape-scale disturbance-succession dynamics resulting from the cumulative effects of management actions undertaken by individual landowners.

During the simulation period, the objective variables were computed each year and retained for calculation of the objectives. In all objectives but the managed area per year, the first 20 years of simulation were discarded to allow for landscape “adaptation” to the new regime.

### Simulation-optimization procedure

For each management strategy, the simulation-optimization procedure was initialized by randomly generating 800 decision variable combinations (a population). Each combination (an individual) was implemented in 24 random landscapes with a similar property area distribution for a period of 100 years. The values of the six objective variables were computed as the maximum value attained in the 24 evaluations, corresponding to the worst result obtained in each of the objectives. Each individual in the current population was ranked according to how well it achieves the optimization objectives relative to the population, with non-dominated individuals assigned Rank 1. Rank 2 individuals are the non-dominated individuals after removing Rank 1 individuals, and so on. Individuals were then chosen randomly to enter the breeding stage of the genetic algorithm, based on both their non-dominated ranking and their uniqueness. These individuals (the parents) were used to generate the next population, either through mutation of the parent vector (i.e. small changes in randomly chosen decision variable values), or through cross-over between two vectors (i.e., exchange of decision variables between two parents). The cycle (generation) was then resumed, by implementing each individual in the simulated landscape, computing the objective variables, evaluating the effectiveness of individuals in the current population, and randomly choosing the parents of the next generation. Mutation and cross-over probabilities were used at their default values (0.2 and 0.7 respectively). The process was repeated until 400 generations were complete, because preliminary analysis based on the Hypervolume Indicator [53] suggested that stabilization was reached after about 100 generations (Figure S2). This indicator provides a measure of the objective space that is dominated by the current Pareto frontier, bounded above by the point corresponding to the maximum (worst) of each objective achieved in any of the generations. Stabilization of these values can be interpreted as algorithm convergence being achieved in terms of objective values. The final output of the search was the approximated non-dominated Pareto frontier, corresponding to the alternative solutions to the optimization problem.

The procedure as described above was introduced by [54] as the Nondominated Sorting Genetic Algorithm II, implemented in the R environment [55] in the package “mco” [56]. This algorithm was considered particularly adequate because it includes a mechanism to avoid crowding of solutions, that is, to maintain diversity among the population of solutions in every iteration. The direct consequence is that, upon finishing, optimal solutions are maximally spread in the objective space, thus covering the widest possible range of situations. This allows a decision-maker to be able to choose the solutions that best suit his needs, by picking the subset that varies within the intervals of the objectives that he considers the most adequate.

Source code for the landscape generator/simulator/analyzer and optimization procedure (C code run from within R) are available (Source Code S1), as well as the optimization results analyzed in this paper.

### Post-processing of optimization results

The Pareto frontier was visualized using pairwise objective scatterplots and level diagrams, where the objective vectors of the optimization solutions are represented in relation to the values of each objective variable [46]. To reduce dimensionality of the objective vectors and thus allow representation in a bi-dimensional plot, each vector was scored with its Euclidean multivariate distance to the theoretical point where all the objectives are at their lowest (or highest) possible value simultaneously (ideal but unachievable solution). Since there was one objective related to cost, two related to biodiversity and three related to fire risk, objectives were range standardized (to 0–1) and then weighted (multiplied by 6, 3 and 2, respectively), prior to distance computation, so that each group of objectives accounted for one third of the overall distance. A level diagram was produced for each objective, with the position of each optimization solution in the Y axis (distance to the ideal point) being constant across diagrams [46]. This is useful for visually comparing the performance of individual solutions in meeting each objective while having a measure of the overall performance of each, but care should be taken since the Y axis involves equal-weighting of the three groups of objectives, thus providing a partial view of the Pareto frontier.

To explore the consequences of decision-maker preferences for achieving landscape level objectives, optimization results were further processed by restricting solutions to those meeting a set of objective constraints, according to what could be considered feasible in practical terms (implementation cost) and reasonable in objective outcomes (fire risk and biodiversity). Restrictions were imposed considering the potential preferences of: a (i) funding agency, limiting management area to 5% or 10% (±0.375%) of the landscape each year; a (ii) forest management agency, requiring that each fire risk objective should lie within the 25% best results observed in simulations; and (iii) a conservation agency, requiring that a minimum area of 10% should be maintained over time for both the 30–60 years and the >60 years age classes; and (iv) a compromise scenario, where all the previous preferences were taken into account, while relaxing the associated constraints. The consequences of these restriction scenarios on the solution space were visualized with pairwise scatterplots of objective values achieved by optimal solutions, to adequately evidence the trade-offs.

In order to analyze in detail the temporal variation in the landscape level objectives resulting from feasible solutions, the management variables of all the compromise solutions (scenario iv) were implemented in random landscapes and simulated during 100 years. The values of the objectives, as well as landscape composition in terms of understory age classes, were then plotted in relation to simulation year. Because the simulator accounts for uncertainty when implementing management, the consequences of each solution were plotted as the mean value assessed through 100 simulations, each in a different random landscape.

### Influence of *a priori* assumptions

To assess the extent to which key *a priori* assumptions and decisions influenced the final results, we conducted two sets of optimization runs with changes in landscape structure and implementation uncertainty. In the first set of runs, we explored the effects of changing the distribution of landholding areas when generating random landscapes, by specifying Gamma distributions with increasing mean and variance values (mean/variance): 20/100, 60/900, 100/2500, 140/4900, 200/10000. In the second set of runs, we explored the effects of changing the uncertainty associated with management intervals when implemented by landowners, by specifying Gamma distributions with increasing maximum variance (that corresponding to the 100-year interval): 1, 5, 10, 50, 100, 500 and 1000.

For each new set of landholding and uncertainty parameters, simulation-optimizations were run for 400 generations, repeating each solution evaluation in 12 random landscapes. Optimization results were then post-processed and compared to those obtained with the base simulation conditions. To help visualize differences between the base solutions and those generated with each level of the tested parameters, we used Principal Coordinate Analysis to represent the pairwise distances between solutions in a 2-dimensional space [57]. To estimate distances, we first specified each solution as a probability density function of management intervals. The density of a solution was estimated with a Gaussian kernel with a smoothing bandwidth fixed at 5, using as weights the proportion of landowners with each management interval. We then computed the intersection (ranging from 0 to 1) between the density functions of each pair of solutions, and used one minus the intersection as a distance metric [58].

## Results

### Overall simulation-optimization results

**Figure 1:**
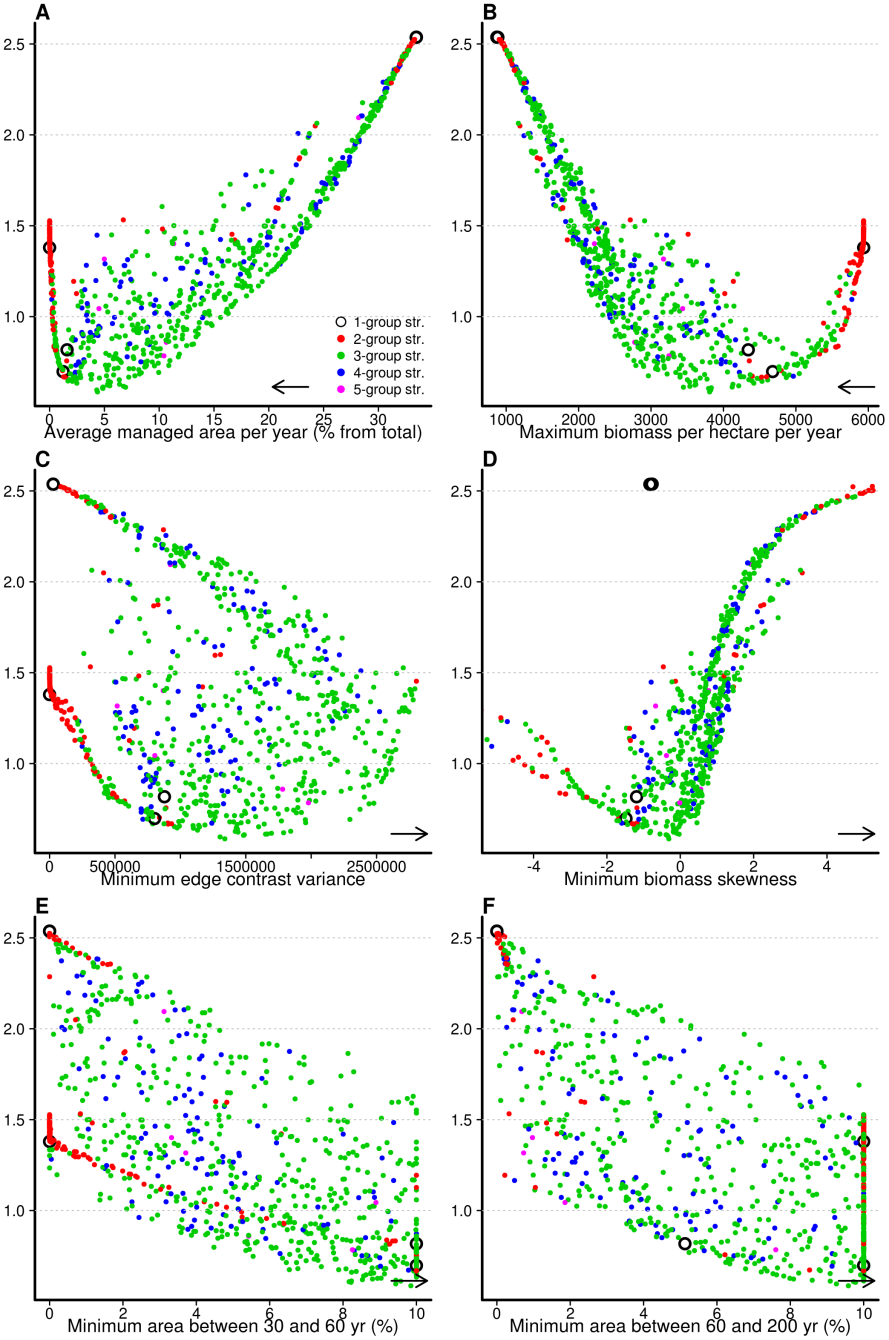
Performance of the optimal management solutions for the five management strategies. Level diagrams showing the objective outcomes of optimal solutions in each of the six objectives (A–F, X axis). Colors correspond to the number of different landowner management groups of each solution. The Y axis (the same across all plots) represents the Euclidean distance to the ideal solution, i.e., a theoretical solution that achieves the best possible values in all objectives simultaneously. Distances were computed giving equal weights to cost (A), fire risk (B–D) and biodiversity (E–F) objectives (see text for details). The arrow in each objective axis point to the direction that is to be achieved during optimization (minimize or maximize).

**Figure 2:**
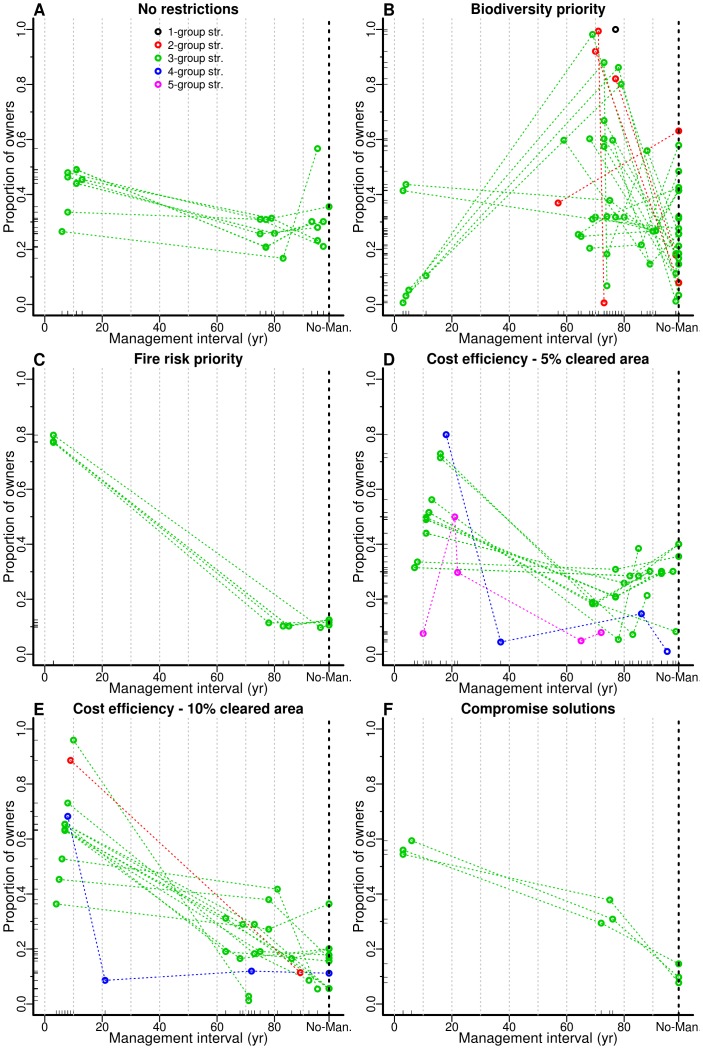
Details of optimal management solutions. Plots represent all the management solutions that fulfill each restriction scenario (B–F, see text for details) and the ten solutions that minimize the Euclidean distance to the ideal solution in the no-restriction scenario (A). Each solution is represented by a set of connected points that describe the management groups that compose the solution. Each point represents a group with a given assigned management interval (X axis) and a given proportion of landowners belonging to it, relative to the total of landowners (Y axis). Colors depict the number of different management groups of each solution. The vertical black dotted line indicates the value interpreted as no-management by the landscape simulator.

The simulation-optimization procedure indicated that multiple group strategies were largely dominant (*sensu* Pareto-dominance) over single group strategies, which comprised only 5 solutions out of 800 (Figure 1). Most non-dominated solutions (ca. 70%) involved three groups of landowners with different management regimes, suggesting that this strategy provided the largest potential and flexibility in fully exploring the feasible objective space. Among these solutions, those that best minimized the distance to the ideal solution, assuming equal-weighing of cost, fire risk and biodiversity objectives (i.e. a no-preference scenario), were characterized by a group with about 40% of landowners clearing the understory at short intervals (ca. 10 years), another group with about 30% clearing with long intervals (75–80 years) and the remaining not managing (Figure 2A). Solutions involving a different number of groups largely maintained the same pattern, with a larger group managing at short intervals and one or more groups managing at different intervals within the 70–100 year time frame (Figure 2A), though they were less satisfactory than the three group strategies in minimizing the distance to the ideal solution. The multiple group solutions with more than three groups tended to converge, along optimization generations, to the three group strategy (plots not shown). It is also noteworthy that there were very few solutions involving landowners managing at 20–60 year intervals, and these mostly belonged to the four group strategy (Figure S3).

The single group strategies always performed poorly in achieving the overall landscape level objectives (Figure 1). Possible solutions included management by all landowners every 3 years, which reduced fire risk, but did not meet cost and biodiversity conservation objectives. In contrast, solutions involving lack of management by all landowners provided a good solution to maintain late successional understories, but failed in the remaining objectives. The third set of possible solutions involved all landowners managing at about 70-year intervals, leading to landscapes that reasonably fulfill both biodiversity objectives, but where the fuel load tends to be too high, and its distribution across the landscape tends to be too continuous, as indicated by the low edge contrast of fuel loads (Figure 1).

### Objective trade-offs in the Pareto frontier

**Figure 3:**
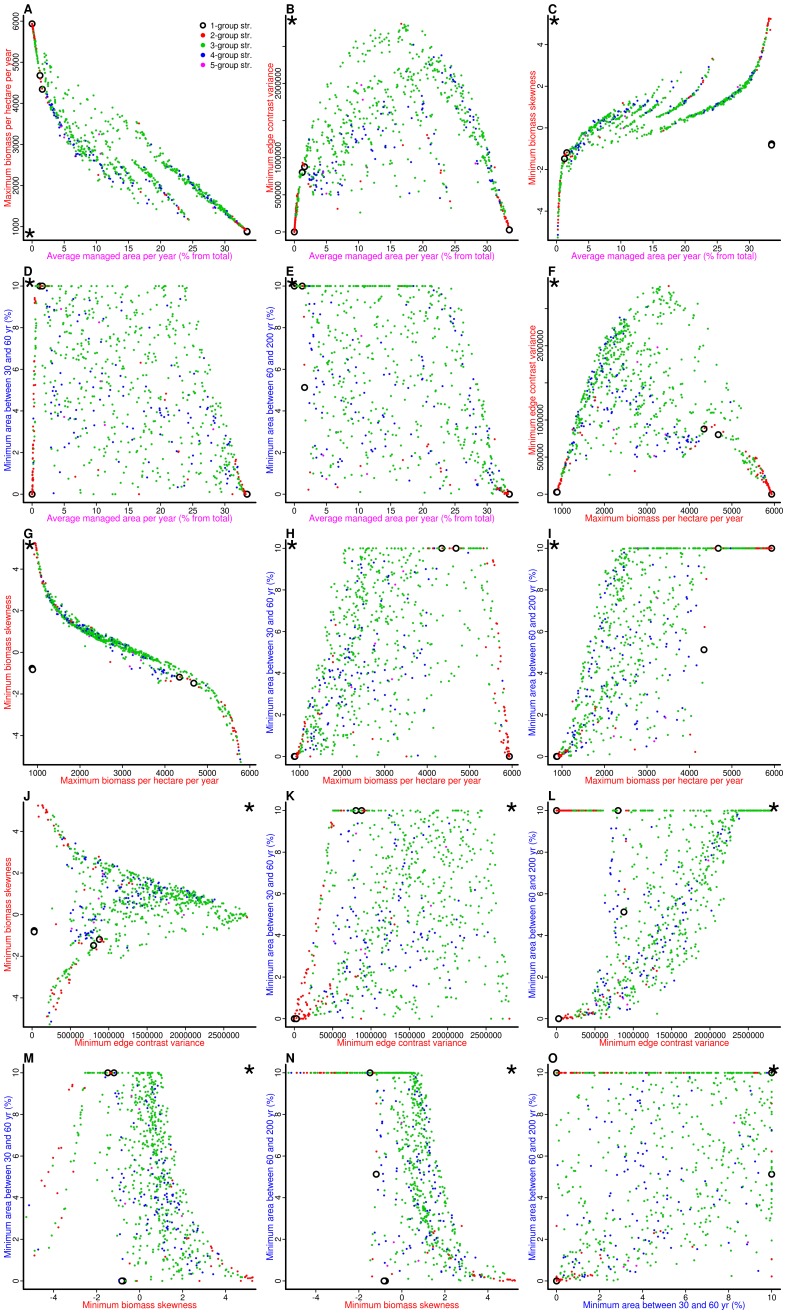
Pairwise objective trade-offs. Scatterplots showing the relationships between the objective outcomes of all the solutions in the Pareto frontier. Colors refer to the number of different management groups of each solution (N = 1 to 5 groups). The asterisk indicates the direction to which the solutions should converge during optimization, i.e., the direction where each pair of objectives is minimized/maximized. For clarity, axis legends are colored according to the subject of each objective: cost (purple), fire risk (red) and biodiversity (blue).

Pairwise objective scatterplots revealed the presence of major trade-offs between some objectives, irrespective of management strategy (Figure 3). Solutions with low fuel loads and high fuel concentration in a few areas always required high proportions of the landscape treated annually (Figure 3A,C), thereby underlining a trade-off between management cost and fire risk reduction. In general, there were also trade-offs between fire risk objectives and the conservation of mid- and late- successional biodiversity, as there was a strong tendency for solutions with low fuel loads and high fuel concentration, also having low proportions of mid- and late-successional understories (Figure 3H,I,M,N).

Multiple group strategies, particularly the strategies involving three groups of landowners with different management regimes, increased the feasible objective space and thus contributed to mitigate the trade-offs of potentially contradictory objectives (Figure 3). For instance, management regimes involving at least three groups of landowners greatly reduced the trade-off between maintaining high edge contrast and the conservation of mid- and late successional understories (Figure 3K,L). The same situation occurs as to reconciling the achievement of biodiversity objectives with low biomass (Figure 3H,I), and high edge contrast with low average managed area (Figure 3B).

### Consequences of management preferences

Constraining the total area managed each year to approximately 5% or 10% of the landscape evidenced trade-offs that were only slight in the unconstrained scenario (Figure S4 and S5). For the 5% constraint, solutions would generally involve a group of 30–80% of landowners clearing the understory at about 7–20 year intervals, and one or more groups managing with different intervals within the 65–100 year time frame (Figure 2). Irrespective of the solution, however, this constraint would imply a high fire risk, due to a high fuel load, high fuel continuity across the landscape and a marked trade-off between the two (Figure S4F). Yet, the objectives for the mid- and late-successional biodiversity could reasonably be achieved without any difficulty in reconciling both (Figure S4O). For the 10% constraint, solutions would involve a group of 40–95% of landowners managing at 5–10 year intervals, and the remaining managing at different intervals within the 60–100 year time frame. This is basically similar to the 5% constraint, with an intensification of management operations of the most frequent group (higher frequency and higher proportion of landowners), and resulted in the same objective trade-offs, albeit closer to the ideal points. Hence, despite this constraint, these solutions would produce reasonable results in terms of fire risk and biodiversity objective variables (Figure S5F,J,O).

If management is focused on fire risk, assuming very ambitious goals for the corresponding objective variables (i.e., the 25% best outcomes for each of the three fire risk objectives), then the only possible solutions – only five – would involve most landowners (about 80%) clearing the understory at very short intervals (3 years), and the remaining divided in two groups, one not managing at all, the other managing with a ca. 80 year interval. Irrespective of the solution, the costs would always be very high, because over 25% of the area would have to be managed annually (Figure S6). Furthermore, the proportion of area occupied by stands with either mid- or late-successional understory would fall below 10% during the simulation period, and thus the biodiversity objectives would never be achieved, especially the mid-successional one.

Focusing on biodiversity objectives provides a large number of alternative solutions, most of which perform very poorly due to its high fire risk (Figure S7). However, some biodiversity solutions are globally similar to some of the best solutions of the no-restriction scenario, involving one group of about 40% of landowners managing at 3–4 year intervals, about 35% managing at about 70 year intervals, and the remaining not managing at all (Figure 2B). It is noteworthy that there are mainly two types of solutions that fulfill biodiversity objectives. The latter, which present a good compromise between biodiversity and fire risk (Figure S7F, small isolated group), and another type based on a marked predominance of long management intervals, which sacrifices all fire risk objectives (Figure S7F,G, large group). Also, it is important to note that a single group strategy (all managing at a 77 year interval) arises as a possible solution fulfilling both biodiversity objectives (Figure 2B).

There was no optimization solution achieving simultaneously all the most ambitious goals in terms of cost, fire risk and biodiversity. However, relaxing these goals to targets of less than 20% of the landscape managed each year, of fire risk objectives within the best 60% obtained in the optimizations, and a minimum representation of 9–10% of mid- and late-successional understory ages, highlighted only three possible solutions, which underlines the difficulties behind reconciling all objectives simultaneously (Figure S8). These solutions were all similar, and involved a group of about 55% of landowners clearing the understory at about 3–6 year intervals, another group of about 35% clearing at a ca.75 interval, and the remaining 10% not clearing at all (Figure 2). These results evidenced the advantage of the three group strategy over the others. Indeed, when relaxing even more the constraints, the dominance of the three group strategy in the solutions was clear (plots not shown).

### Landscape dynamics under optimal management

**Figure 4:**
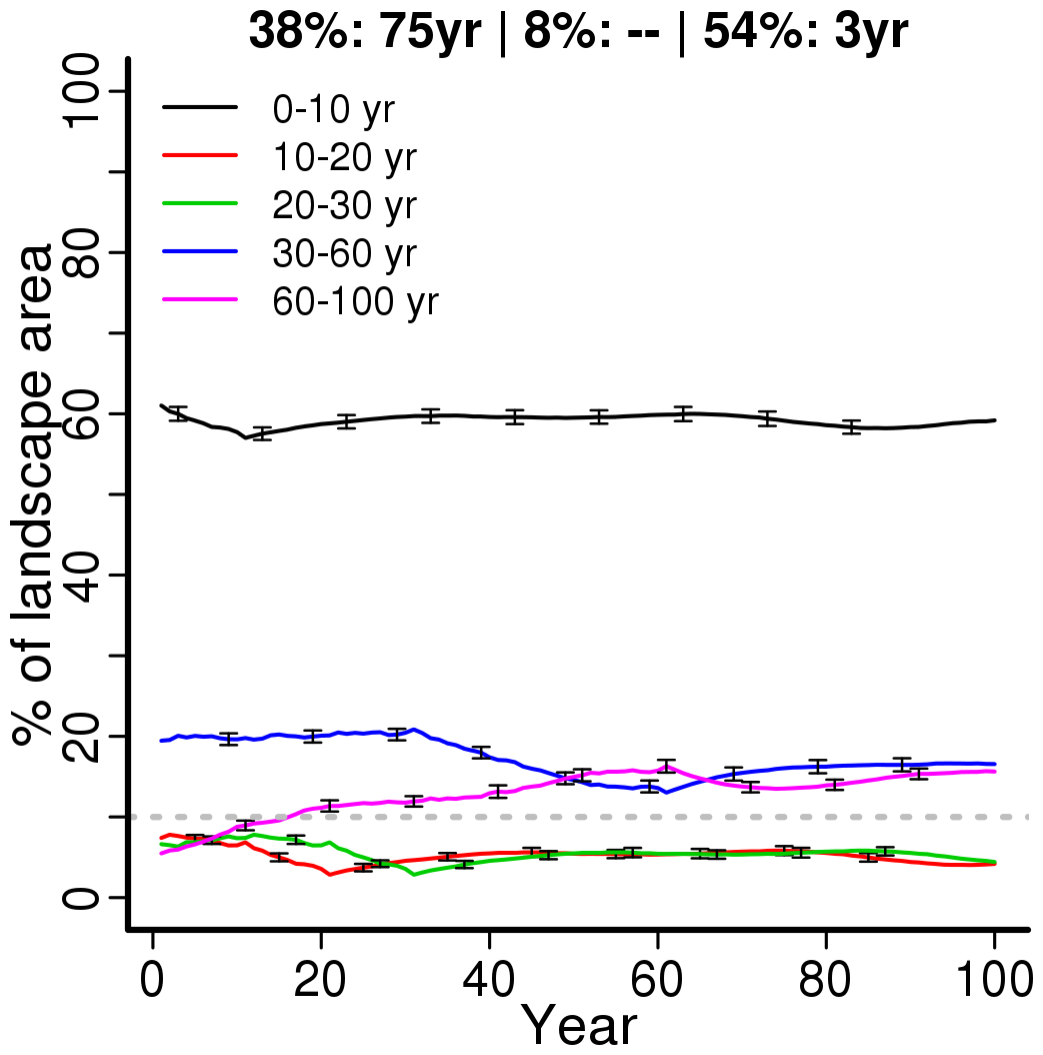
Dynamics of a landscape managed according to a compromise solution. Percentage area (Y axis) of landscape in each age class throughout the simulation period (X axis) in landscapes managed according to one of the regimes obtained in the compromise solution scenario (Figure S8). The other two compromise solutions (very similar) were omitted for simplicity. Lines depict the mean values (±standard deviations) across 100 simulations in different random landscapes subjected to the management regime.

**Figure 5:**
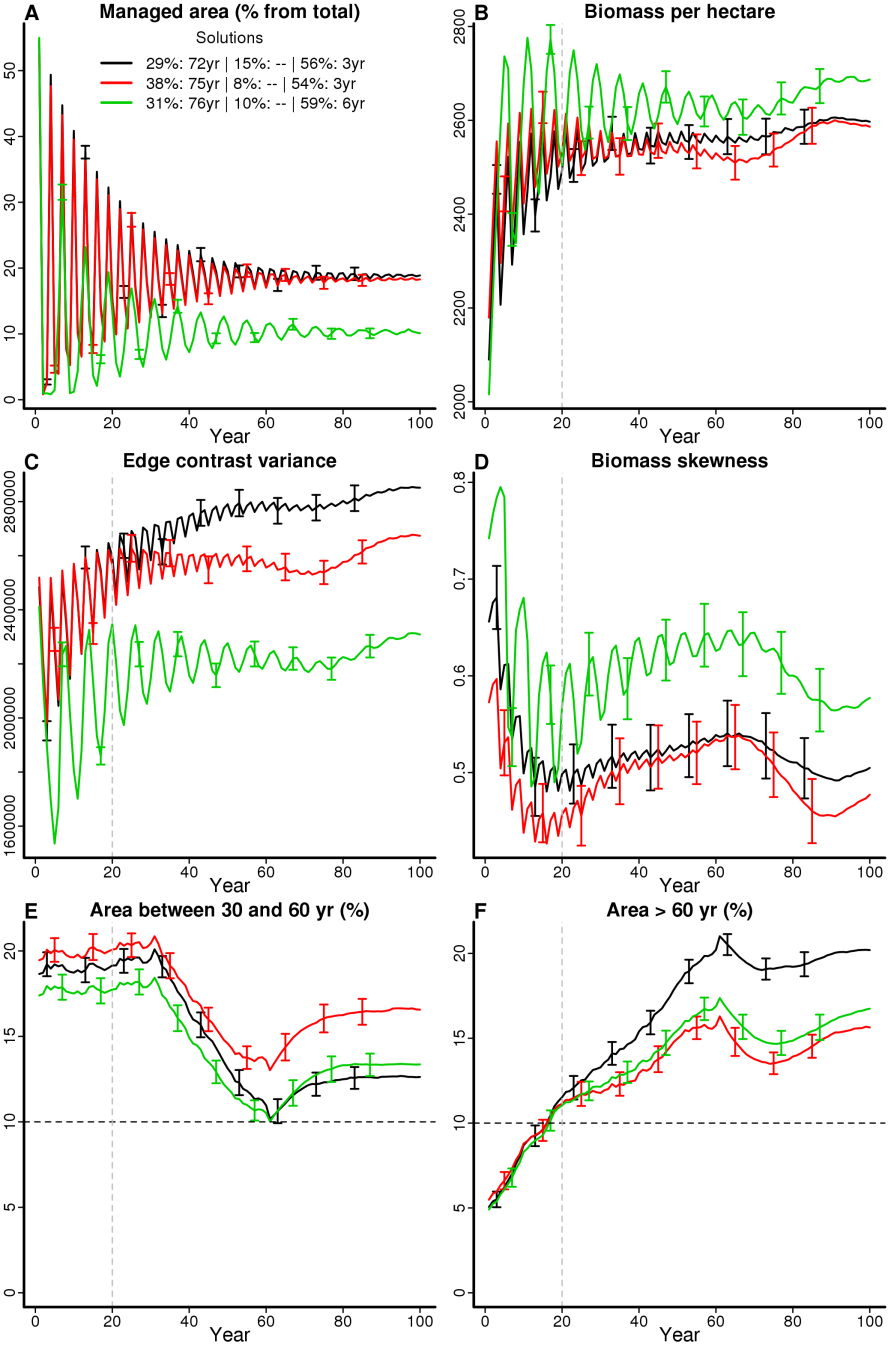
Objective dynamics in a landscape managed according to compromise solutions. Plots showing the values taken by the six objectives (A–F) along the simulation period (X axis) of the three compromise solutions (Figure S8). Each line corresponds to a solution and depicts the mean values (±standard deviations) across 100 simulations of different random landscapes subjected to the management regime of the solution. Objective values used during the optimization algorithm correspond to the average (A) or the minimum (B–F) taken along the whole simulation period (A) or discarding the first 20 years (B–F, vertical dashed line).

The three compromise solutions resulted in a landscape largely dominated (ca. 60%) by stands with young understory ages (<10 years), whereas both mid- (30–60 years) and late-successional (>60 years) understories were represented by about 10–20% of the landscape (Figure 4). This pattern of landscape composition was nearly the same for all compromise solutions (plots not shown). Trends in cost and fire risk management objectives showed a cyclic component, with large fluctuations in the first decades and a subsequent convergence to rather stable values (Figure 5). The proportion of mid-successional ages showed a large decline towards the mid of the simulation period, with a fast recovery thereafter (Figure 5). This effect would become increasingly less pronounced (i.e. the local minima would increase) if the simulations were run longer than 100 years (plots not shown). The proportion of late-successional ages, on the opposite, increased gradually until nearly stabilizing about 60 years after the start of simulation, and would show a similar trend as the previous in the longer term.

### Influence of a priori assumptions

The Principal Coordinates (PCo) biplots accounted for a large proportion of variation in the data (ca. 70–95%; Figure S9 and S10), indicating that they provide adequate summary representations of distances between different solutions. In general, PCo biplots suggested that distances between solutions did not show any important systematic effect of changing the distribution of landholding areas (Figure S9). In fact, for each restriction scenario, there was great overlap in the solutions obtained with the base and the alternative simulation conditions, albeit less so in the cost efficiency scenario (10% cleared area). Visualization of the Pareto frontier using pairwise objective scatterplots and level diagrams (not shown), also suggested that optimization results were largely consistent irrespective of changes in the distribution of landholding areas.

Variation in the level of uncertainty in management implementation appeared to have a stronger effect than landholding area in the optimization solutions, and this was most evident in the cost efficiency and compromise restriction scenarios (Figure S10). In both cases, the solutions optimized under high uncertainty tended to cluster apart from the remaining solutions (Figure S10C, E). In contrast, solutions optimized under moderate and low uncertainty did not show any obvious differences from the main optimization run (corresponding to uncertainty = 100). In terms of dominant strategies, as uncertainty increased, optimal solutions under no restriction scenarios tended to be dominated by the two group strategy (plots not shown), while on the opposite extreme (intervals implemented nearly exactly as prescribed), a wide range of strategies and distinct solutions arose. As to the objectives, there were only very slight differences in the objectives achieved by optimal solutions along the uncertainty gradient (plots not shown).

## Discussion

Designing management strategies to secure the services provided by landscapes made up of patchworks of private properties is challenging, due to the inherent stochasticity in landowners' responses to management rules, which in turn result in temporal and spatial variations in landscape composition and configuration that are hard to predict. The multi-objective simulation-optimization approach described in this study provides a tool to deal with these challenges, by explicitly integrating uncertainty in the implementation of management rules by individual landowners. Furthermore, this approach based on Pareto optimality provides a range of potential solutions to each optimization problem, allowing detailed examination of trade-offs between management objectives and identification of the consequences of management preferences by decision makers (e.g. [12]). Although the approach was described using a particular case, it may be sufficiently flexible to deal with other socio-ecological systems where the provision of landscape services results from the cumulative effects of individual decisions by multiple landowners (e.g. [4]). It is thus expected that this approach may find wide applicability to help solve management problems in multi-ownership landscapes.

### Management strategies for upland cork oak landscapes

**Figure 6:**
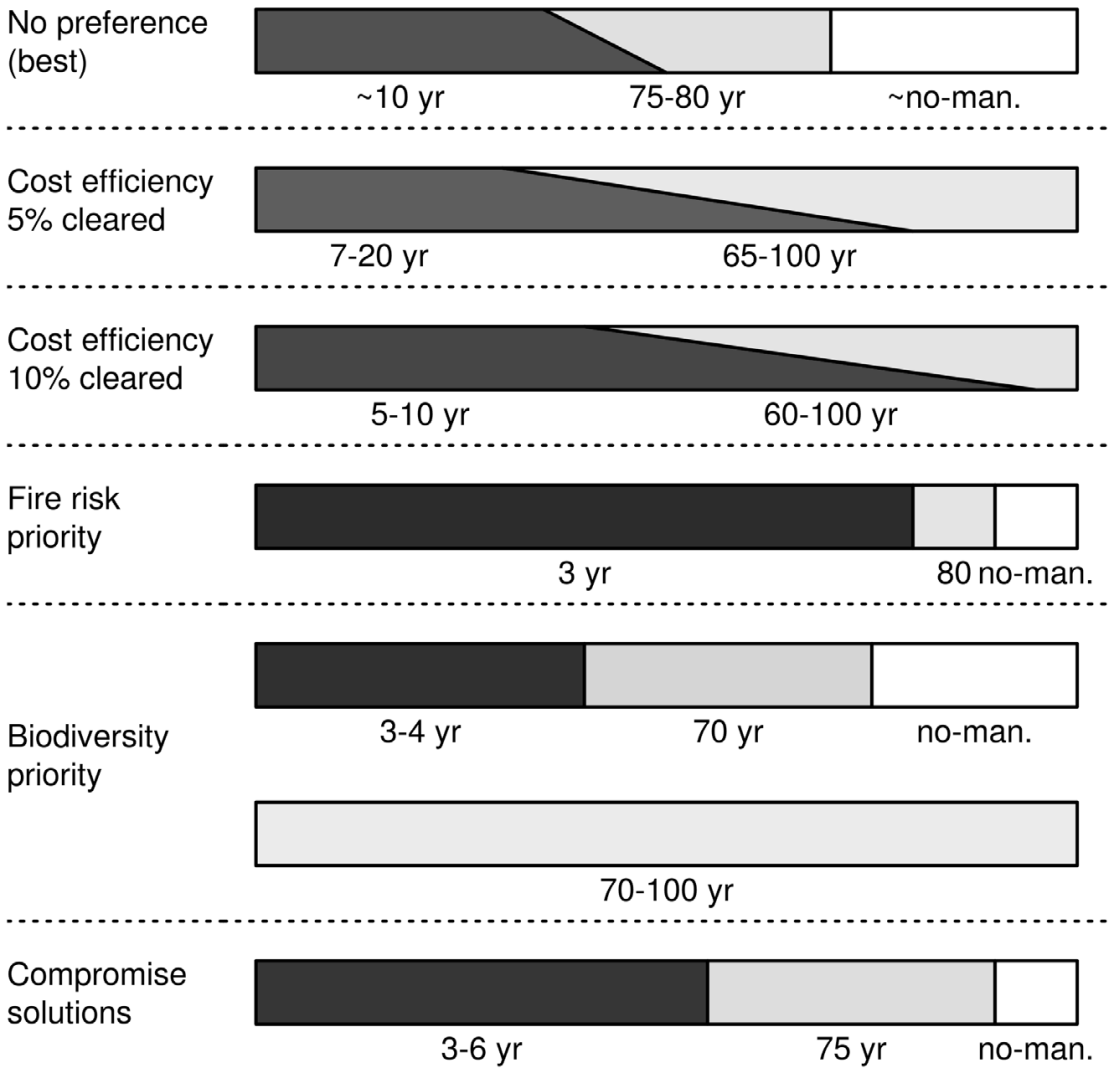
Summary of optimal management solutions in each restriction scenario. Each bar represents the approximate general composition of all the solutions fulfilling each restriction scenario, and of those ten that minimize the distance to the ideal solution in the no-restriction scenario. Each segment (within a bar) represents one group of landowners, whose proportion is given by segment length. Shade intensity is proportional to the average management interval of the respective group (indicated below). Groups that are flexible in terms of proportion of owners are represented with oblique boundaries extending between the approximate minimum and maximum allowable proportions.

Despite a number of simplifications and assumptions taken in the development of the simulation-optimization approach for the management of cork oak upland landscapes, the study provided valuable guidelines for reducing fire risk and conserving mid- and late-successional biodiversity, while controlling for management costs. These guidelines, summarized in Figure 6, should be taken as tentative, because the development of detailed management recommendations would require the incorporation of a great deal of additional realism in the landscape simulation conditions and a better understanding of the uncertainties associated with management implementation.

A key result of the simulation-optimization is that there should be two or more groups of landowners, each associated with a given management regime, whose cumulative action contributes to achieving the landscape-level objectives (Figure 6). Most management solutions involved a group of about 50%–90% of landowners clearing the understory at short intervals (3–9 years), though the exact proportions and intervals depended on management preferences. If reducing fire risk was the overarching goal then the percentage of landowners in the short rotation cycle was highest and the management interval was about 3–4 years, whereas a focus on biodiversity yielded the lowest proportion of landowners in the short rotation cycle (null in some cases) and management intervals of up to 11 years. Irrespective of the details of the management regime, however, there was a very clear indication that a large proportion (>50%) of the landscape needs to be recurrently managed if the accumulation of fuel load is to be prevented. These management guidelines clearly contrast with current practice, though traditional understory clearing in association with the 9-year cork extraction cycle is within the optimal management intervals emerging from this study. However, ongoing processes of rural depopulation and land abandonment have resulted in a declining proportion of the landscape managed with this rotation cycle, which thus results in over-accumulation of biomass and increasing fire risk [26], [39], [59], [60].

Besides the short rotation cycle, most management solutions also involved two other groups of landowners, one clearing the understory with a long rotation cycle (about 70–90 years), and the other not clearing at all. In contrast, very few solutions involved management at intervals of about 20–60 years. The proportion of landowners associated with the long rotation cycle is largest if management focuses on biodiversity conservation, and smallest (but not null) if the main focus is fire risk reduction. The emergence of long rotation and no-management schedules in the optimization process is probably a consequence of the biodiversity objectives, being necessary to maintain a sufficient representation of stands with mid- and late-successional understory. Irrespective of the management details, the key result is that areas with old understory can only be maintained if there is a distinct group of landowners that are required to clear the understory at long intervals or even not manage at all. As in the case of the short rotation cycle, these management guidelines contrast with current practice, due to uncoordinated management among landowners. In these circumstances, mid- and late-successional understories are cleared at irregular intervals, and so they may disappear from the landscape if the average recurrence interval is less than about 30 and 60 years, respectively.

Combining a large proportion of landowners under the short rotation cycle regime, with two smaller groups under the long rotation and no-management schedules, results in a landscape with low fire risk while maintaining biodiversity associated with mid- and late-successional understories. The implementation of this management regime would result in a heterogeneous landscape dominated by a patchwork of stands with herbaceous or small shrubland understory, though maintaining a reasonable representation of patches with complex and multi-layered understory [36]. This landscape would likely contribute for the conservation of Mediterranean forest biodiversity, by providing conditions for a wide range of species associated with early, mid and late successional habitats [35]–[38]. Furthermore, this mosaic landscape would be suitable for endangered species requiring complementary habitats, including for instance top predators that breed or shelter in undisturbed stands with late-successional understories, while feeding on prey such as rabbits (*Oryctolagus cuniculus*) that are associated with patchworks of shrublands and herbaceous habitats [61]–[63]. The simulation-optimization procedure thus converged to a management regime promoting landscape heterogeneity, which is an overarching goal in forest landscapes [64], [65].

### Improving the simulation-optimization approach

While the simulation-optimization approach developed in this study was useful to generate insights on the optimal management of upland cork oak landscapes, its use for guiding the actual management of real landscapes would require introducing far more realism in landscape conditions and simulation parameters. One key limitation was that initial simulation conditions were based on virtual landscapes, rather than in an actual landscape. Although this was necessary due to the shortage of information on the boundaries of private properties and on the initial distribution of understory ages, it should be stressed that making detailed management prescriptions would require simulations based on the actual characteristics of a real landscape [66]. Another problem was that the simulated landscapes assumed spatial and temporal homogeneity of environmental conditions, though this was a simplification since natural heterogeneities associated with, for instance, slope, exposure, soil type, and temporal climatic changes, may strongly influence vegetation successional pathways, biomass accumulation and management costs [35], [36]. Furthermore, in this study we have only considered a biomass accumulation curve of fine fuels and leaves, though there may be some differences in the accumulation of different fuel types (e.g. coarse and overall fuels), with consequences for fire risk [39]. All or part of these additional complexities could be easily incorporated in the landscape simulation algorithm, depending on the amount of ecological data available. This would require the association of management units with a set of environmental variables, which could then be used to modify biomass accumulation curves and management cost as a function of relevant environmental conditions [43], [60]. Undertaking these exercises was beyond the scope of the present study, due to limitations in the information available.

In contrast to most other studies dealing with the optimization of landscapes to increase fire resistance, the decision variables used in optimization were not spatially explicit, which may be regarded as a shortcoming of our approach. Indeed, while these types of studies normally focus on the optimal spatial distribution of fuel treatments (e.g. [5], [11], [12]), our approach dealt with the temporal distribution of fuel treatments by groups of landowners, without considering explicitly any spatial component. In fact, the spatial component in our procedure was specified only in terms of landscape configuration objectives, by optimizing variables related to spatial fuel continuity and concentration. This option was purposefully taken to better incorporate the inherently stochastic character of decisions by landowners in multi-ownership landscapes, where it is nearly impossible to guarantee that a given treatment will be allocated to a given spatial location. Because of this, our approach produced solutions indicating, for instance, that there should be three groups of landowners involved in either short rotation, long rotation or no management schedules, but not where in the landscape these differential management regimes should be better assigned. Future research efforts should be devoted to extend our simulation-optimization approach in order to consider decision variables with both temporal and spatial components, introducing uncertainty associated with both the timing and the location of fuel treatments. A further related improvement would be to scrutinize the uncertainty “black box” by better understanding how landowners respond to various factors, instead of just using random uncertainty to account for all influent factors that are unknown. This need is highlighted, for instance, by the influence of changes in uncertainty on the simulation results. Devising the rules that drive landowner responses, in order to turn the landscape simulator into an agent-based model, could be guided, for example, by insights gained from participatory simulations involving real landowners, which would further approximate the model to the real world situation.

Biodiversity objectives considered in this study were very simple, specifying only that a given minimum percentage of mid- and late-successional habitats should be retained in the landscape over 100 years. More complex goals could be incorporated within our framework by using species-specific habitat and/or metapopulation models to estimate variation in species diversity and abundance over the simulation period (e.g. [4], [22], [67]), and then specifying optimization objectives such as the maximization of species diversity or habitat quantity for particular species or groups of species. This procedure could be particularly useful to design landscapes for species of conservation concern (e.g. [12], [18]), in the context of multi-objective optimization and duly accounting for stochasticity inherent to multi-ownership landscapes. Combining our simulation-optimization approach with species-specific habitat models will be the subject of future research.

### Optimizing the management of multi-ownership landscapes

The approach to the management planning of multi-ownership landscapes developed in this study differed in a number of significant ways and it is expected to overcome some of the practical problems associated with previous approaches. In the first place, previous approaches assumed that there is an optimal landscape configuration, but give little consideration on how such landscape can be produced in practice, assuming that there are no impediments to implementation [68], [69]. Secondly, they assume that the optimal configuration is static and can be maintained through a spatial and temporal scheduling of management activities rigorously implemented by individual landowners (e.g. [18]), failing to account for landscape dynamics [33], [68], [69]. Finally, they assume, often implicitly, that there is coordination among landowners in the implementation of the management plan (e.g. [18], [68]). These assumptions make it doubtful whether these approaches can find wide applicability in real landscapes, because management rules are often poorly enforced and thus may not be strictly respected by landowners [23]. Furthermore, allocating specific management regimes to particular spatial locations may have high social and economic costs, which may be hard to support [30], [43], [68], [70].

To solve previous limitations, our approach was based on the idea that landscape-level objectives can emerge from uncoordinated and uncertain responses of individual landowners to management rules, without explicit spatial planning [4], [30]. This led to the development of a simulation-optimization approach that incorporates some of the complexities and uncertainties associated with the management of real landscapes, recognizing that a static landscape configuration may never be achieved due to the inherent stochasticity in landowners' decisions and their imperfect compliance with management rules as well as to natural succession. Despite these problems associated with the management of multi-ownership landscapes, our approach was able to show that landscape-level objectives, similarly to what [71] suggest, can indeed be obtained by defining simple, non-spatial management rules that are implemented individually by each landowner, subject to uncertainty, and without the need for coordination among them. The approach provided dynamic landscape configurations that optimized the landscape-level objectives, without requiring a precise spatial allocation of management activities. The mechanism for producing such optimal landscapes is inherent in the simulation-optimization procedure, because the optimization of management rules is conditional on the degree to which they achieve the landscape-level objectives from the beginning to the end of the simulation period.

The extent to which our approach can be applicable to other socio-ecological systems is uncertain, but it is likely that it could provide valuable insights for the management of a range of different multi-ownership landscapes. In particular, the approach may be useful where management involves the creation and maintenance of spatial heterogeneity in ecological conditions, which is often a key management goal in a range of forest [64], [65], [72] and agricultural [42], [73] landscapes. There are cases, however, where the application of this approach may be inappropriate, requiring a more conventional spatially explicit landscape planning (e.g. [18]). This may be the case, for instance, where the conservation of biodiversity or environmental functions is strongly associated with particular locations, and so there is no flexibility in the spatial allocation of management regimes. Exploring the limits of applicability of the approach outlined in here, as well as eventual improvements that may make it more widely applicable, should be the subject of future research. Comparative evaluation of the cost-effectiveness of spatial versus non-spatial solutions to a wide range of management problems could prove particularly valuable.

## Supporting Information

Figure S1 **Implementation uncertainty at each management interval.** Boxplot representations (median, quartiles and extremes) of the distributions used to map theoretical management intervals (as proposed in the solutions) to real-world management intervals (as implemented in practice in the simulations), in the main optimization run. Each boxplot represents 10000 random values drawn from a Gamma distribution with the mean equal to the X axis, and variance computed as a function of the mean (see text for details).
(TIF)

Figure S2 **Convergence of the optimization algorithm.** Convergence was assessed by the hypervolume (in the objective space) that is dominated by the current Pareto front at each generation (X axis) of the main optimization run.
(TIF)

Figure S3 **Details of all optimal management solutions.** Plots representing management groups of all 800 optimal management solutions, as a function of assigned management interval (X axis) and proportion of landowners in the group (Y axis). Each point represents a group, and is colored according to the strategy of the solution it belongs to (i.e. the number of different management groups of the respective solution). For the sake of clarity, points of the same solution are not connected. The vertical black dotted line indicates the value interpreted as no-management by the landscape simulator.
(TIF)

Figure S4 **Pairwise objective trade-offs of solutions that result in approximately 5% of the landscape cleared annually.** Scatterplots showing the pairwise relationships between objectives achieved by all solutions in the Pareto frontier. Solutions that have approximately the same implementation cost (A) falling within 5%±0.375% (dashed lines) are highlighted. Colors refer to the number of different management groups of each highlighted solution (N = 3 to 5 groups). The asterisk indicates the direction to which the solutions should converge during optimization, i.e., the direction where each pair of objectives is minimized/maximized. For clarity, axis legends are colored according to the subject of each objective: cost (purple), fire risk (red) and biodiversity (blue).
(TIF)

Figure S5 **Pairwise objective trade-offs of solutions that result in approximately 10% of the landscape cleared annually.** Scatterplots showing the relationships between all pairs of objectives achieved by all the solutions in the Pareto frontier. Solutions that have approximately the same implementation cost (A) falling within 10%±0.375% (dashed lines) are highlighted. Colors refer to the number of different management groups of each highlighted solution (N = 2 to 4 groups). The asterisk indicates the direction to which the solutions should converge during optimization, i.e., the direction where each pair of objectives is minimized/maximized. For clarity, axis legends are colored according to the subject of each objective: cost (purple), fire risk (red) and biodiversity (blue).
(TIF)

Figure S6 **Pairwise objective trade-offs of solutions that best meet fire risk objectives.** Scatterplots showing the relationships between all pairs of objectives achieved by all the solutions in the Pareto frontier. Solutions that best fulfill the three fire risk objectives simultaneously (F, G), i.e., those that fall below the 25% percentile of maximum biomass per hectare per year (e.g. X axis in F–I) and above the 75% percentile in the other two (e.g. Y axis in F, G) are highlighted. Percentiles are depicted by dashed lines. All highlighted solutions belong to the three group strategy. The asterisk indicates the direction to which the solutions should converge during optimization, i.e., the direction where each pair of objectives is minimized/maximized. For clarity, axis legends are colored according to the subject of each objective: cost (purple), fire risk (red) and biodiversity (blue).
(TIF)

Figure S7 **Pairwise objective trade-offs of solutions that fulfill biodiversity objectives.** Scatterplots showing the relationships between all pairs of objectives achieved by all the solutions in the Pareto frontier. Solutions that fulfill both biodiversity objectives simultaneously (O), i.e., resulting at least in 9.9% (dashed lines) of the area maintained in each age class throughout the simulation period, are highlighted. Colors refer to the strategy of each highlighted solution (N = 1 to 3 groups). The asterisk indicates the direction to which the solutions should converge during optimization, i.e., the direction where each pair of objectives is minimized/maximized. For clarity, axis legends are colored according to the subject of each objective: cost (purple), fire risk (red) and biodiversity (blue).
(TIF)

Figure S8 **Pairwise objective trade-offs of solutions that perform reasonably in all objectives simultaneously.** Scatterplots showing the relationships between all pairs of objectives achieved by all the solutions in the Pareto frontier. Solutions that perform reasonably in all objectives simultaneously, i.e., that fall within the unshaded quarter of each plot, are highlighted. Dashed lines correspond to 20% of landscape managed each year (A, X axis), the percentiles 60 (F, X axis), 40 and 40 (F, G, Y axis) of the fire risk objectives, and a minimum of 9% of the area maintained in the two age classes (O, both axis). All highlighted solutions belong to the three group strategy. The asterisk indicates the direction to which the solutions should converge during optimization, i.e., the direction where each pair of objectives is minimized/maximized. For clarity, axis legends are colored according to the subject of each objective: cost (purple), fire risk (red) and biodiversity (blue).
(TIF)

Figure S9 **Influence of landholding area distribution on the optimal solutions found in each restriction scenario (A–E).** Each point corresponds to a solution. Solutions were mapped into a 2-dimensional plot by Principal Coordinate Analysis of a distance matrix computed from the similarity between the density of all pairs of solutions (see text for details). Since original distances are preserved, scale was kept constant across plots to allow direct comparison. Colors refer to the values taken by mean and variance of the Gamma distribution used to sample landholding areas when generating random landscapes.
(TIF)

Figure S10 **Influence of implementation uncertainty on the optimal solutions found in each restriction scenario (A–E).** Each point corresponds to a solution. Solutions were mapped into a 2-dimensional plot by Principal Coordinate Analysis of a distance matrix computed from the similarity between the density of all pairs of solutions (see text for details). Since original distances are preserved, scale was kept constant across plots to allow direct comparison. Colors refer to the values taken by the maximum variance of the Gamma distribution used to assign management intervals to landowners (see text for details). Higher values mean higher uncertainty assumed in the simulations as to the timing of management operations.
(TIF)

Source Code S1 **Source code (R and C languages) of the simulation-optimization algorithms.** All the code for the simulation-optimization algorithms was written within the original source code of the “mco” R package [56], for performance reasons.
(ZIP)
