# Supplementary figures and images for: Optimization of Landscape Services under Uncoordinated Management by Multiple Landowners
Source: PLoS One. 2014 Jan 17;9(1):e86001. doi: 10.1371/journal.pone.0086001 (PMC3895036; doi:10.1371/journal.pone.0086001)

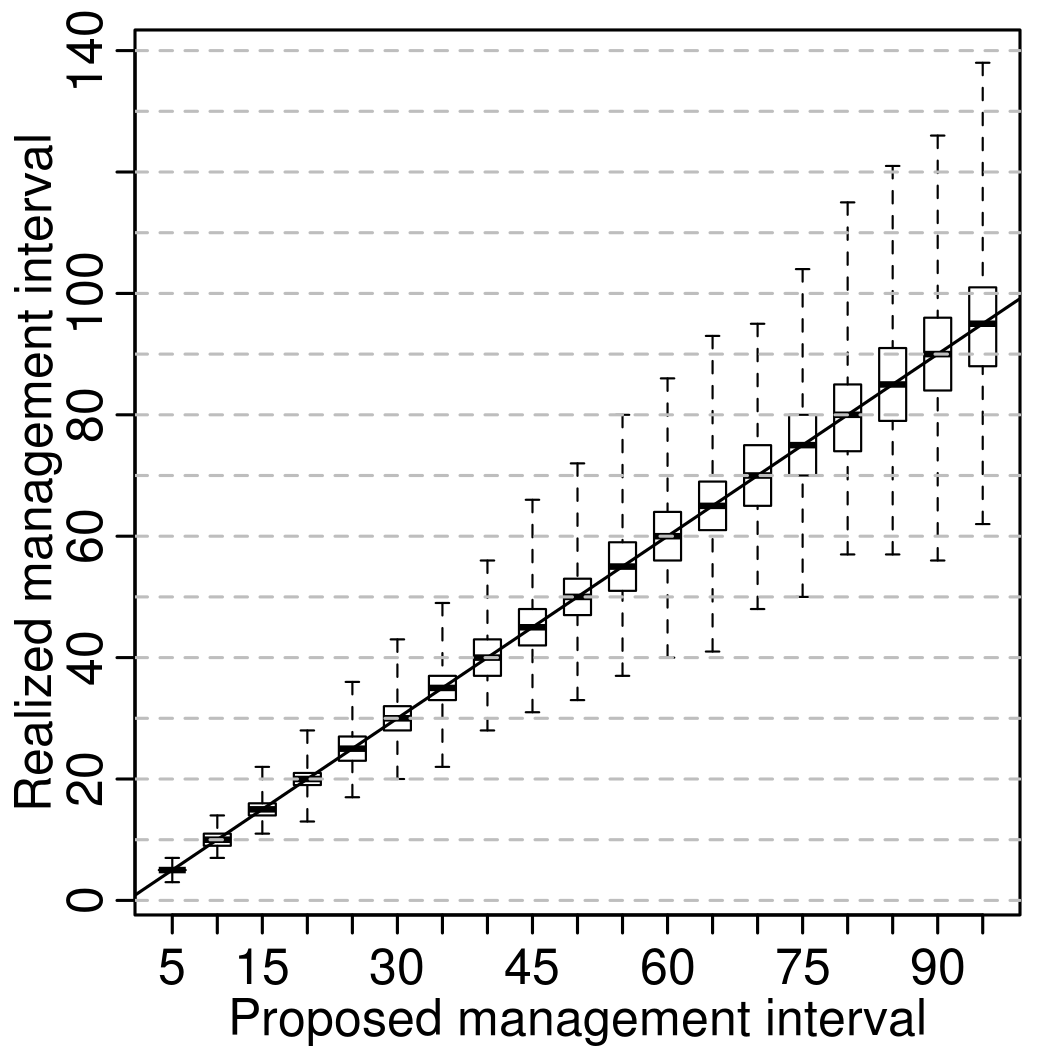

Supplement: Figure S1 — Implementation uncertainty at each management interval. Boxplot representations (median, quartiles and extremes) of the distributions used to map theoretical management intervals (as proposed in the solutions) to real-world management intervals (as implemented in practice in the simulations), in the main optimization run. Each boxplot represents 10000 random values drawn from a Gamma distribution with the mean equal to the X axis, and variance computed as a function of the mean (see text for details). (TIF) [file pone.0086001.s001.tif]

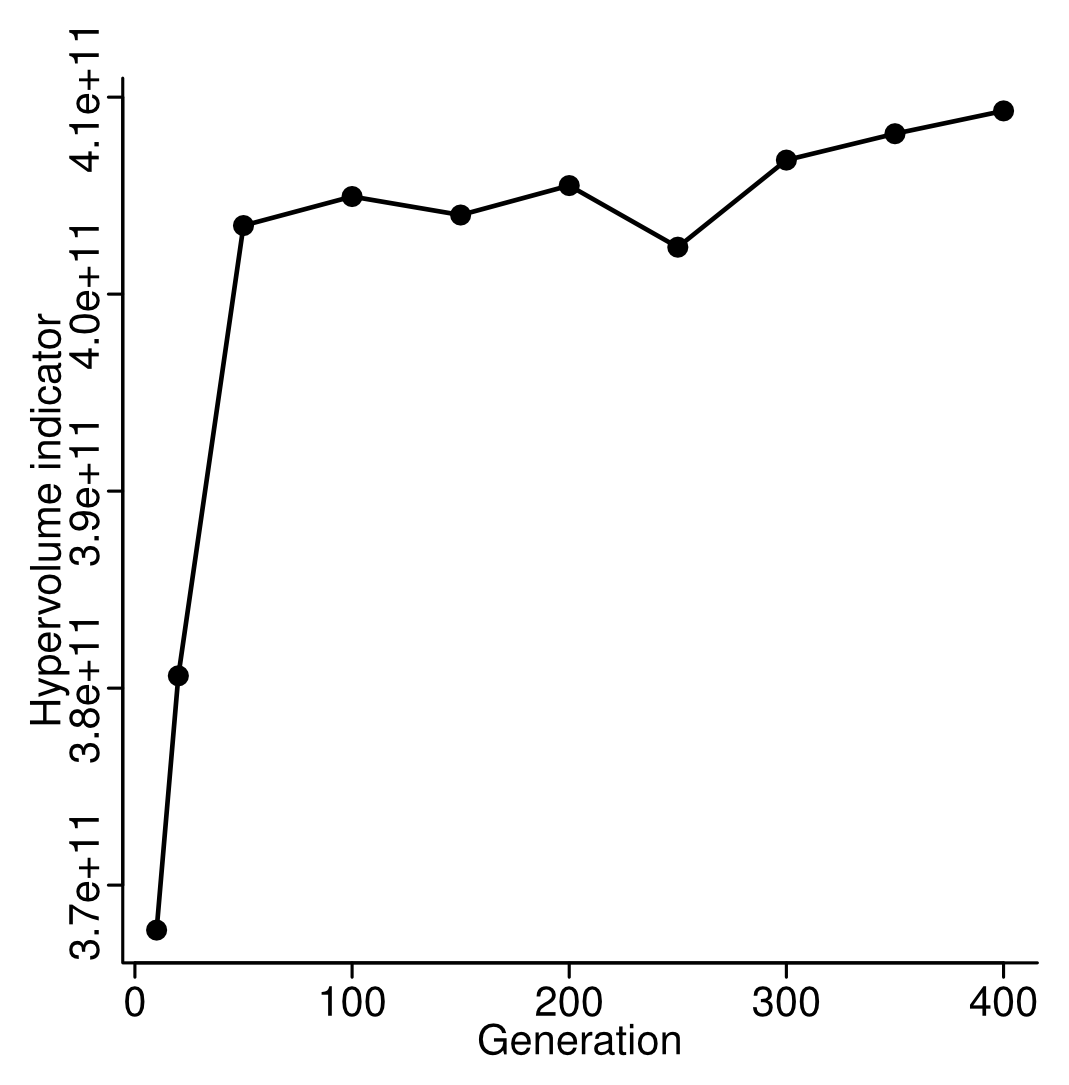

Supplement: Figure S2 — Convergence of the optimization algorithm. Convergence was assessed by the hypervolume (in the objective space) that is dominated by the current Pareto front at each generation (X axis) of the main optimization run. (TIF) [file pone.0086001.s002.tif]

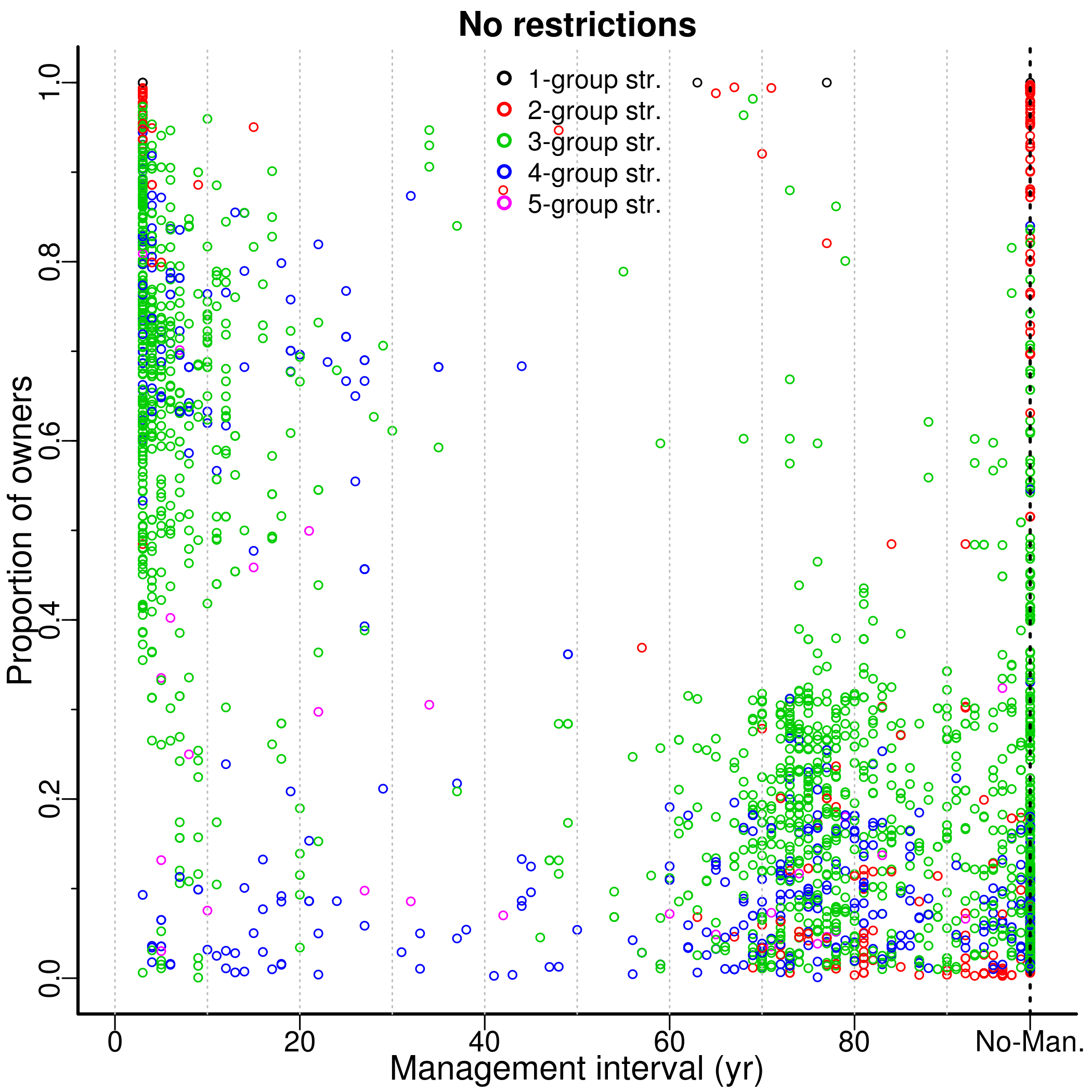

Supplement: Figure S3 — Details of all optimal management solutions. Plots representing management groups of all 800 optimal management solutions, as a function of assigned management interval (X axis) and proportion of landowners in the group (Y axis). Each point represents a group, and is colored according to the strategy of the solution it belongs to (i.e. the number of different management groups of the respective solution). For the sake of clarity, points of the same solution are not connected. The vertical black dotted line indicates the value interpreted as no-management by the landscape simulator. (TIF) [file pone.0086001.s003.tif]

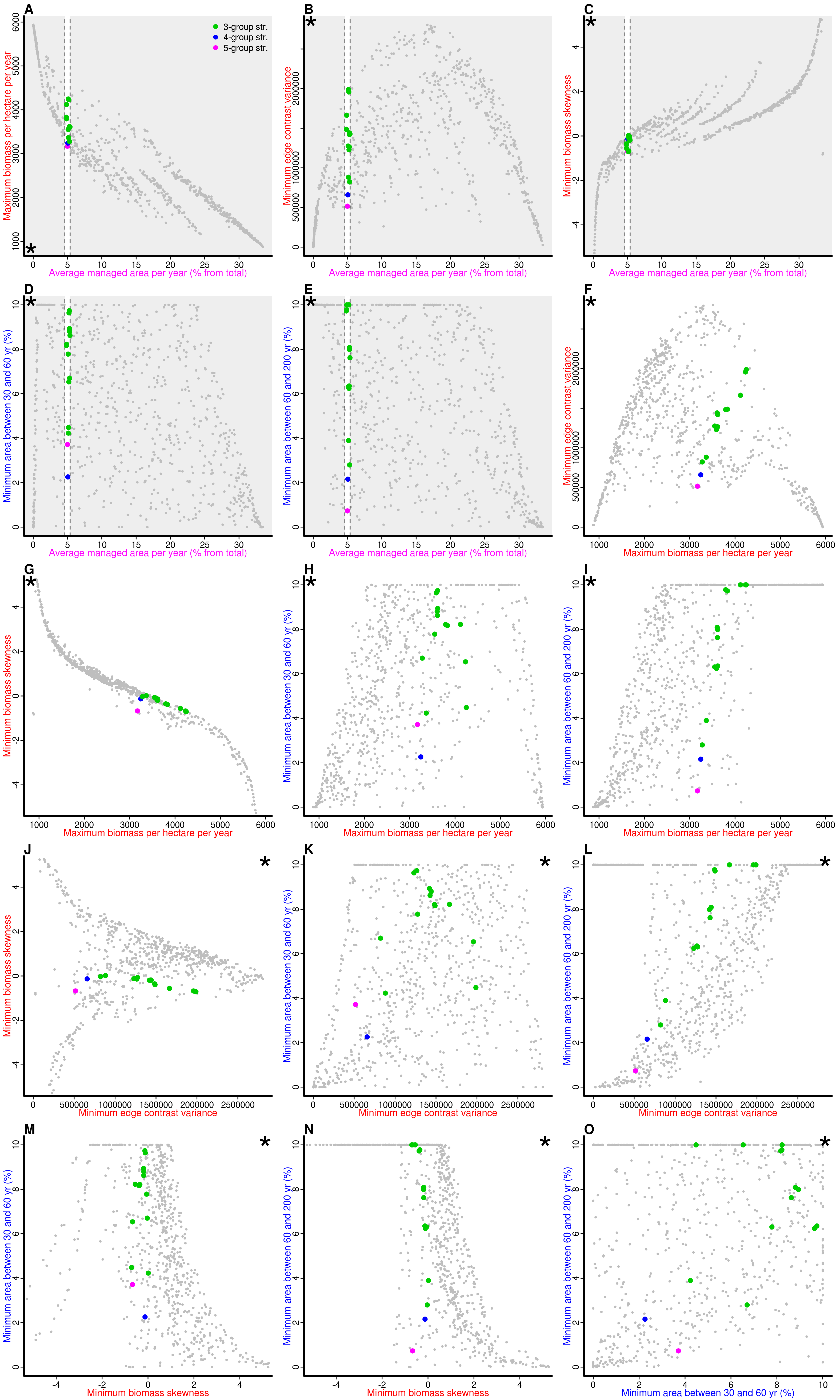

Supplement: Figure S4 — Pairwise objective trade-offs of solutions that result in approximately 5% of the landscape cleared annually. Scatterplots showing the pairwise relationships between objectives achieved by all solutions in the Pareto frontier. Solutions that have approximately the same implementation cost (A) falling within 5%±0.375% (dashed lines) are highlighted. Colors refer to the number of different management groups of each highlighted solution (N = 3 to 5 groups). The asterisk indicates the direction to which the solutions should converge during optimization, i.e., the direction where each pair of objectives is minimized/maximized. For clarity, axis legends are colored according to the subject of each objective: cost (purple), fire risk (red) and biodiversity (blue). (TIF) [file pone.0086001.s004.tif]

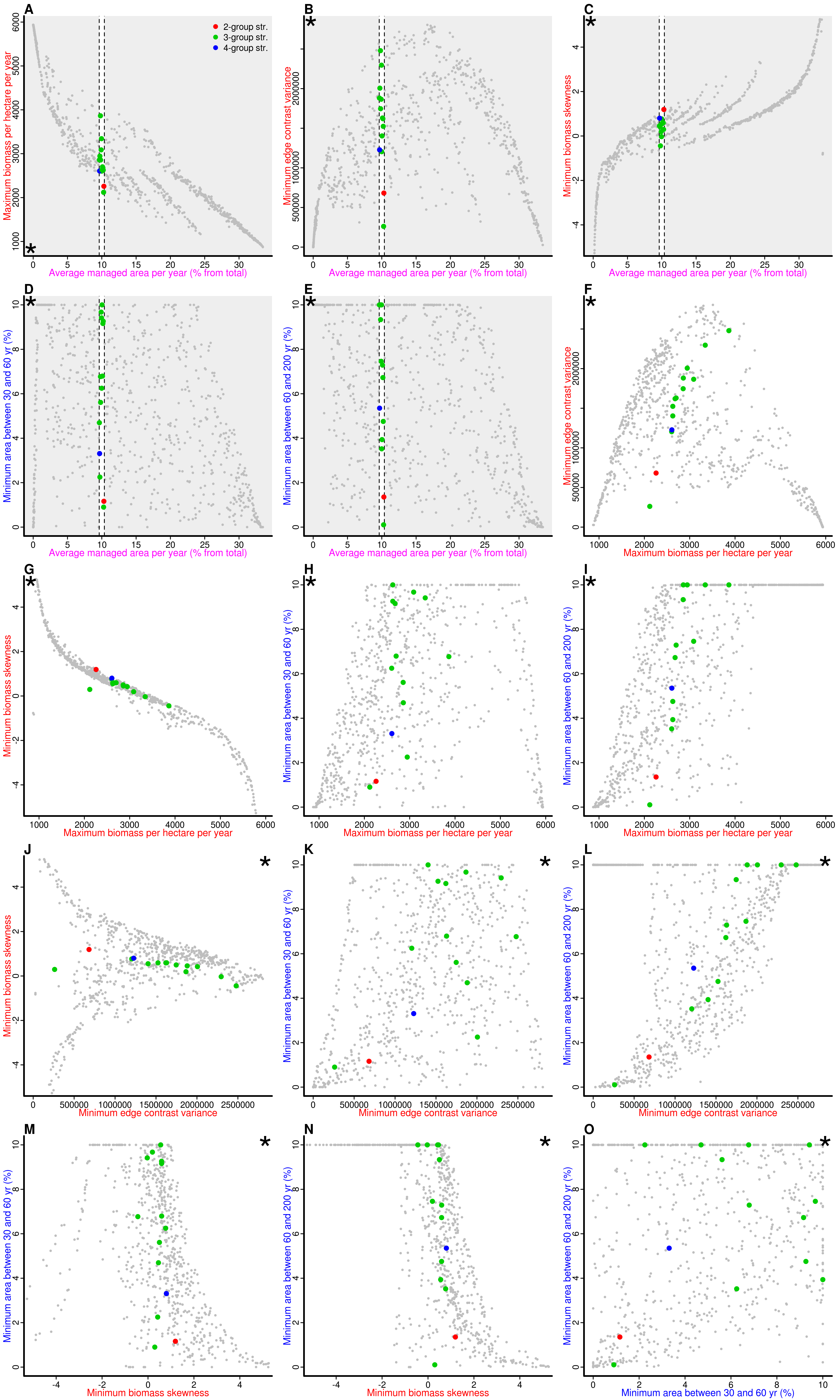

Supplement: Figure S5 — Pairwise objective trade-offs of solutions that result in approximately 10% of the landscape cleared annually. Scatterplots showing the relationships between all pairs of objectives achieved by all the solutions in the Pareto frontier. Solutions that have approximately the same implementation cost (A) falling within 10%±0.375% (dashed lines) are highlighted. Colors refer to the number of different management groups of each highlighted solution (N = 2 to 4 groups). The asterisk indicates the direction to which the solutions should converge during optimization, i.e., the direction where each pair of objectives is minimized/maximized. For clarity, axis legends are colored according to the subject of each objective: cost (purple), fire risk (red) and biodiversity (blue). (TIF) [file pone.0086001.s005.tif]

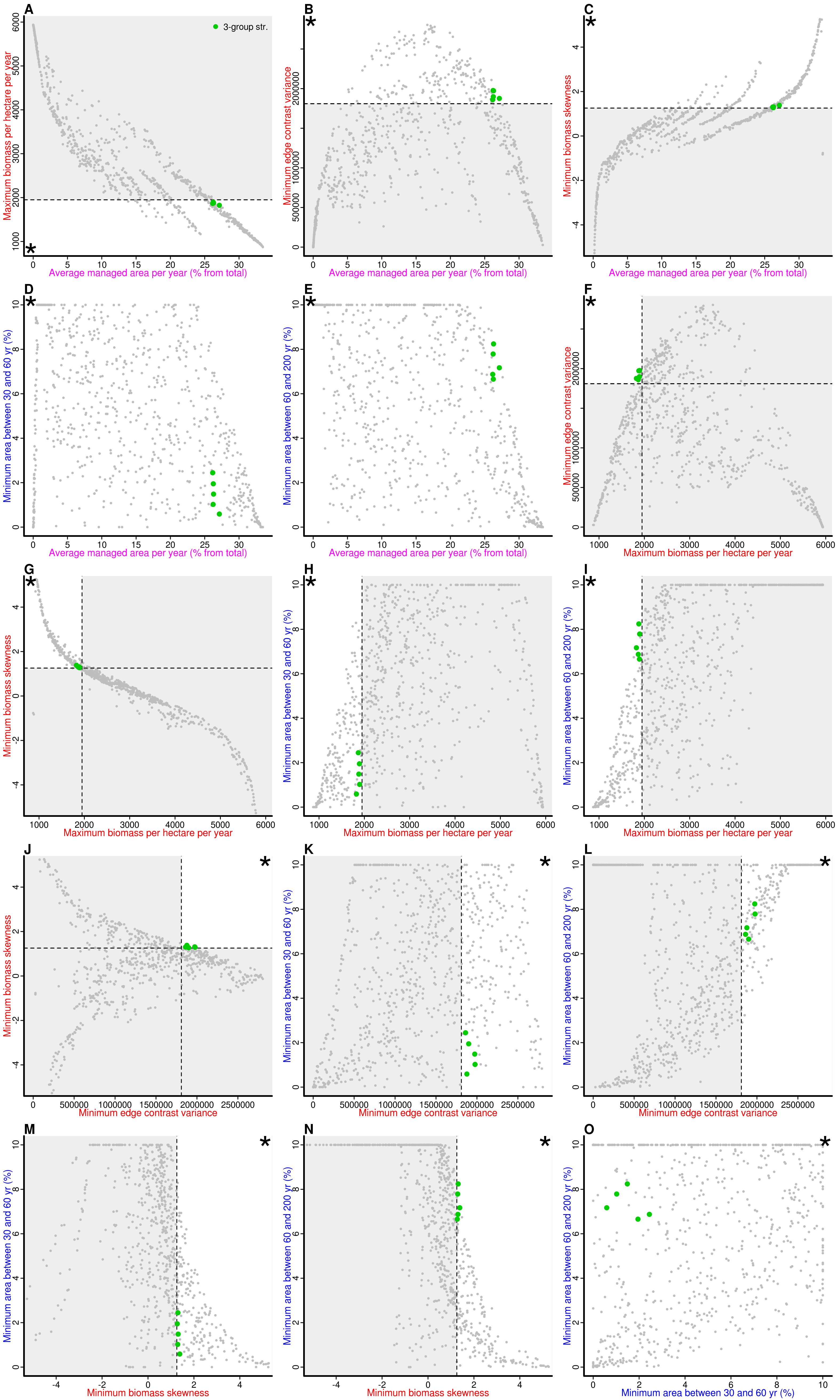

Supplement: Figure S6 — Pairwise objective trade-offs of solutions that best meet fire risk objectives. Scatterplots showing the relationships between all pairs of objectives achieved by all the solutions in the Pareto frontier. Solutions that best fulfill the three fire risk objectives simultaneously (F, G), i.e., those that fall below the 25% percentile of maximum biomass per hectare per year (e.g. X axis in F–I) and above the 75% percentile in the other two (e.g. Y axis in F, G) are highlighted. Percentiles are depicted by dashed lines. All highlighted solutions belong to the three group strategy. The asterisk indicates the direction to which the solutions should converge during optimization, i.e., the direction where each pair of objectives is minimized/maximized. For clarity, axis legends are colored according to the subject of each objective: cost (purple), fire risk (red) and biodiversity (blue). (TIF) [file pone.0086001.s006.tif]

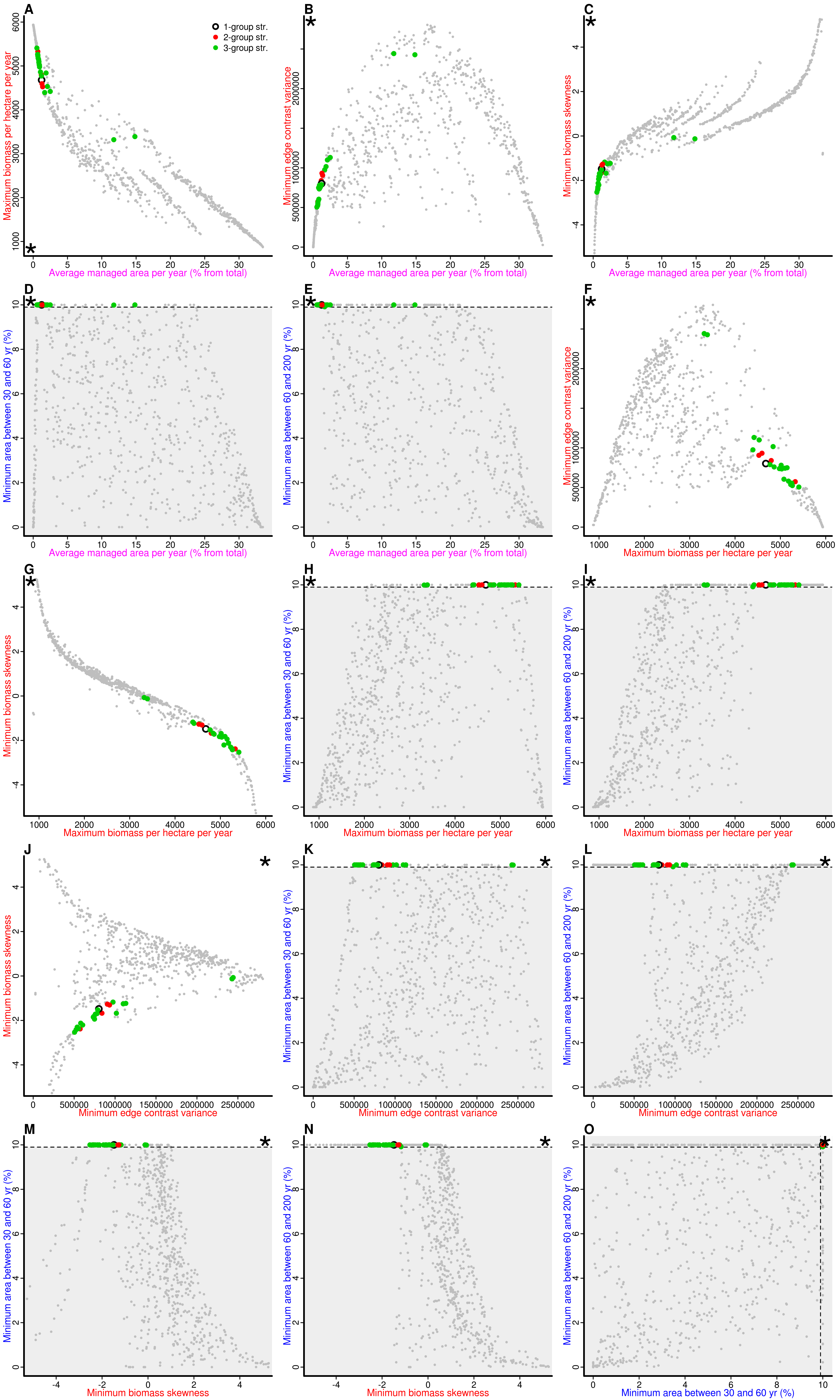

Supplement: Figure S7 — Pairwise objective trade-offs of solutions that fulfill biodiversity objectives. Scatterplots showing the relationships between all pairs of objectives achieved by all the solutions in the Pareto frontier. Solutions that fulfill both biodiversity objectives simultaneously (O), i.e., resulting at least in 9.9% (dashed lines) of the area maintained in each age class throughout the simulation period, are highlighted. Colors refer to the strategy of each highlighted solution (N = 1 to 3 groups). The asterisk indicates the direction to which the solutions should converge during optimization, i.e., the direction where each pair of objectives is minimized/maximized. For clarity, axis legends are colored according to the subject of each objective: cost (purple), fire risk (red) and biodiversity (blue). (TIF) [file pone.0086001.s007.tif]

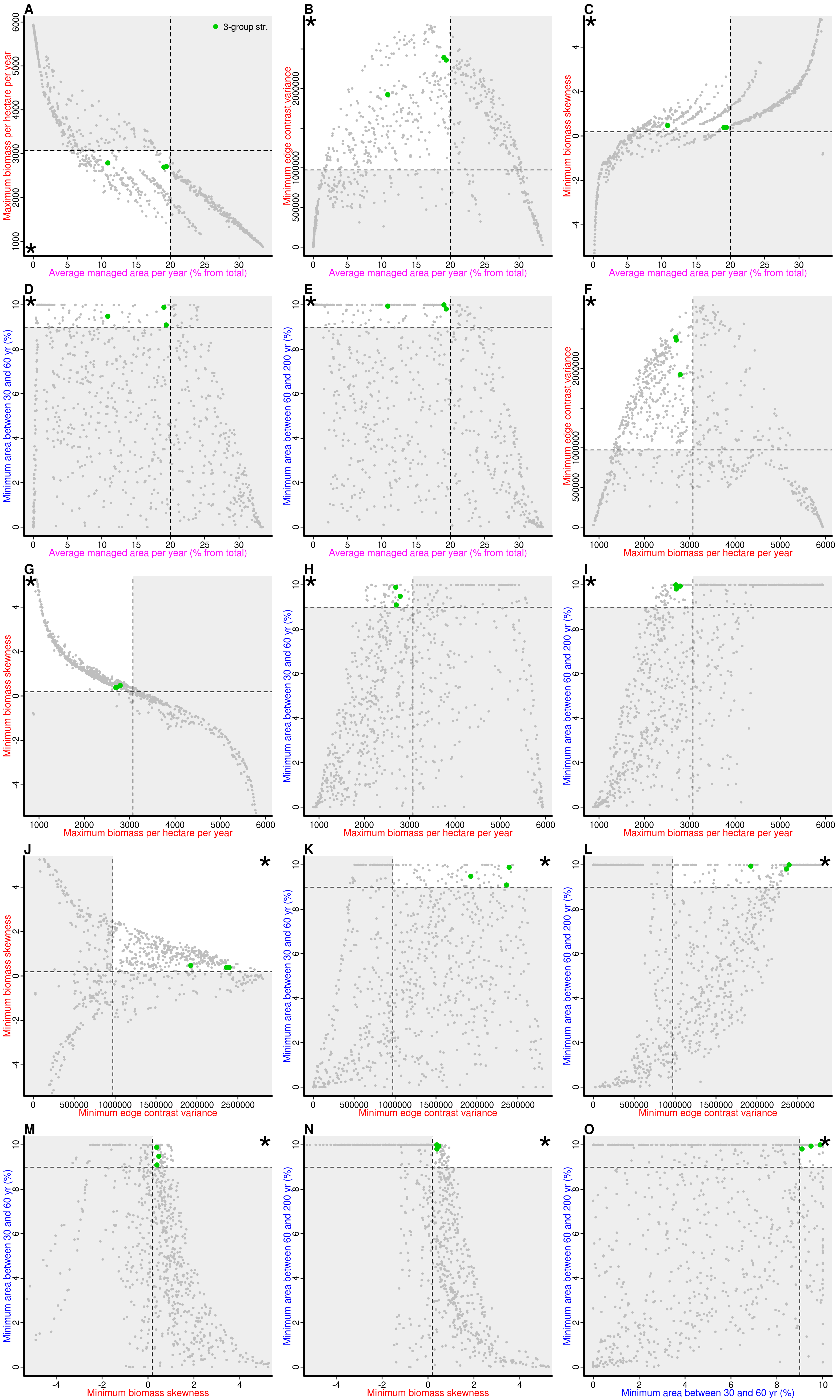

Supplement: Figure S8 — Pairwise objective trade-offs of solutions that perform reasonably in all objectives simultaneously. Scatterplots showing the relationships between all pairs of objectives achieved by all the solutions in the Pareto frontier. Solutions that perform reasonably in all objectives simultaneously, i.e., that fall within the unshaded quarter of each plot, are highlighted. Dashed lines correspond to 20% of landscape managed each year (A, X axis), the percentiles 60 (F, X axis), 40 and 40 (F, G, Y axis) of the fire risk objectives, and a minimum of 9% of the area maintained in the two age classes (O, both axis). All highlighted solutions belong to the three group strategy. The asterisk indicates the direction to which the solutions should converge during optimization, i.e., the direction where each pair of objectives is minimized/maximized. For clarity, axis legends are colored according to the subject of each objective: cost (purple), fire risk (red) and biodiversity (blue). (TIF) [file pone.0086001.s008.tif]

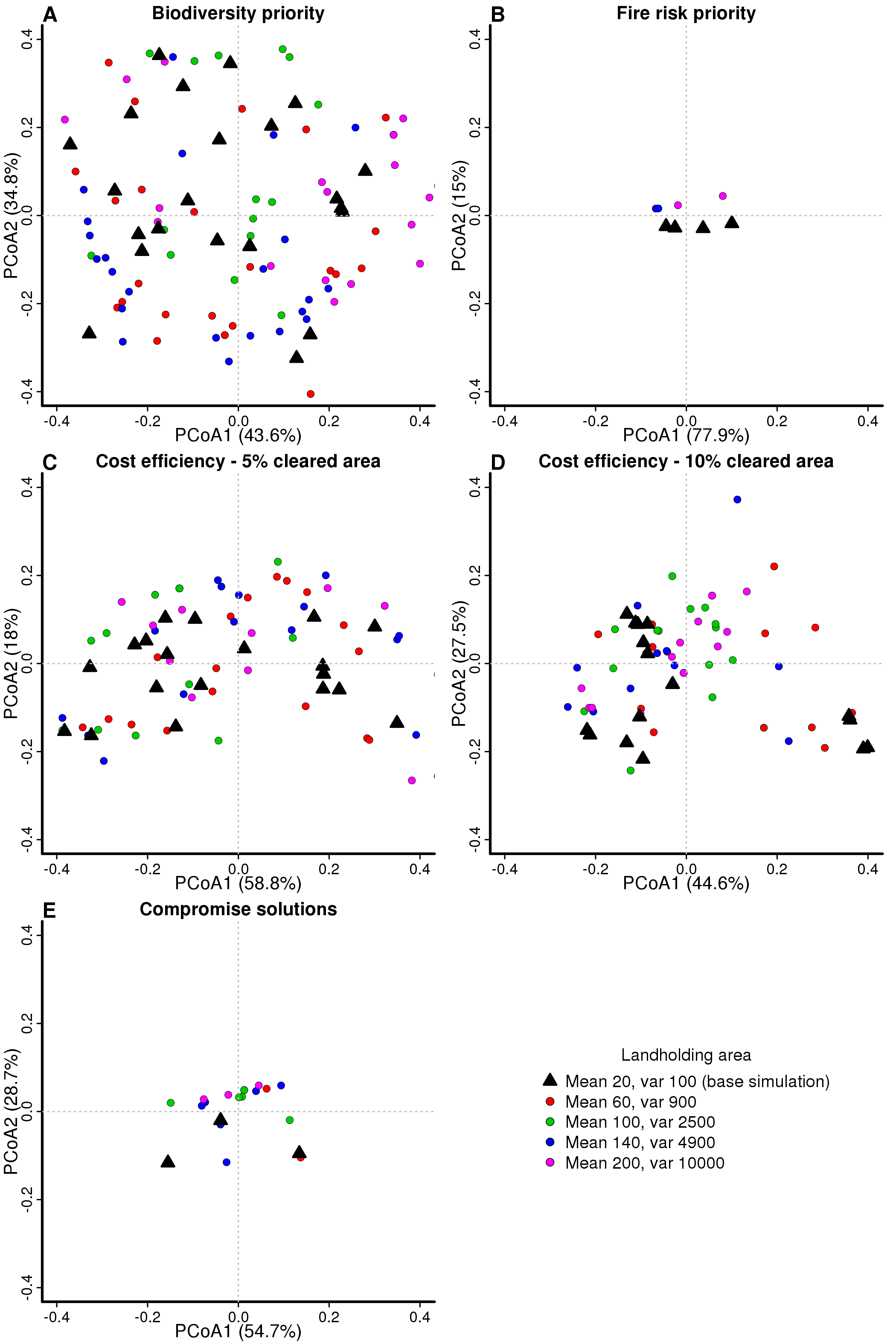

Supplement: Figure S9 — Influence of landholding area distribution on the optimal solutions found in each restriction scenario (A–E). Each point corresponds to a solution. Solutions were mapped into a 2-dimensional plot by Principal Coordinate Analysis of a distance matrix computed from the similarity between the density of all pairs of solutions (see text for details). Since original distances are preserved, scale was kept constant across plots to allow direct comparison. Colors refer to the values taken by mean and variance of the Gamma distribution used to sample landholding areas when generating random landscapes. (TIF) [file pone.0086001.s009.tif]

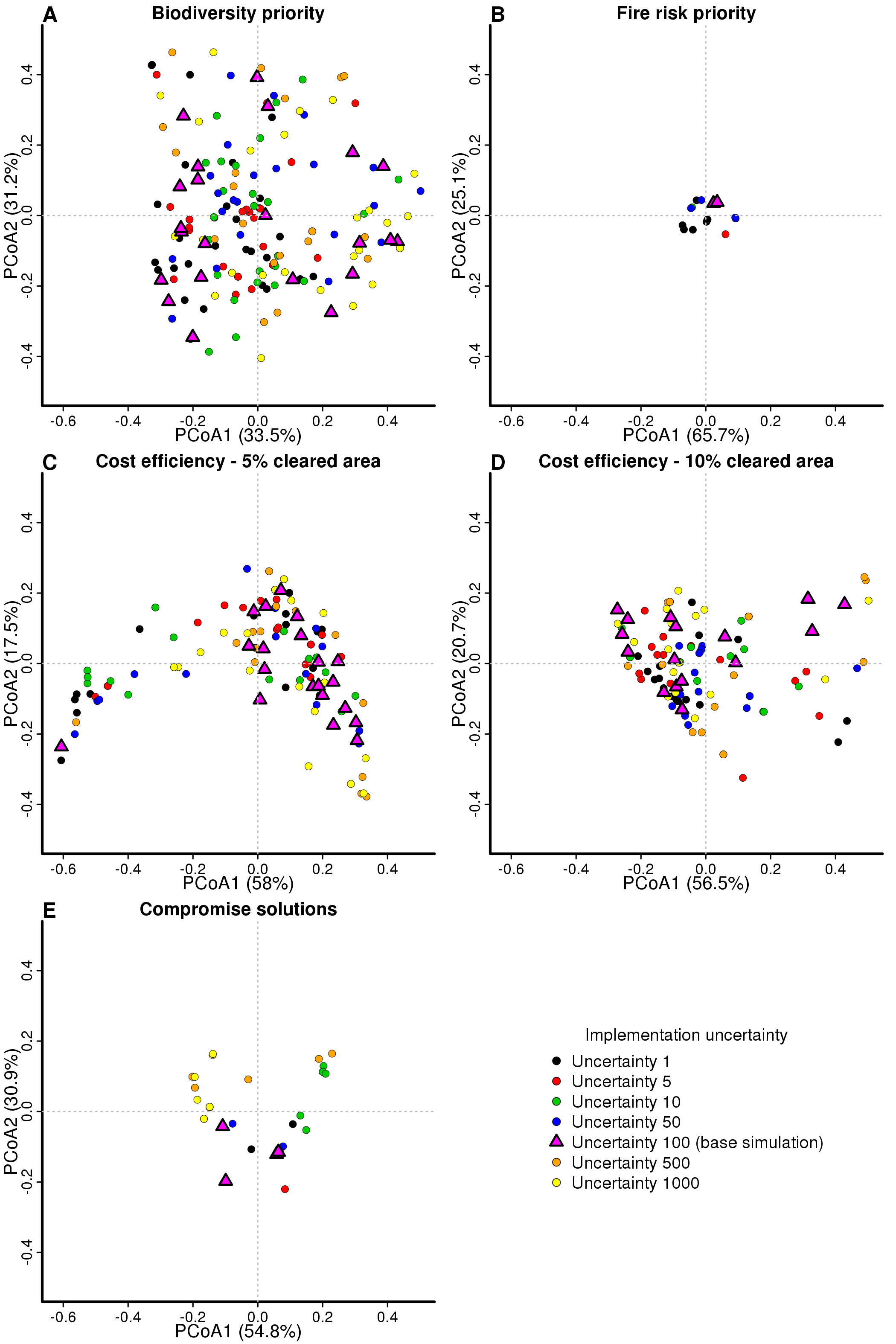

Supplement: Figure S10 — Influence of implementation uncertainty on the optimal solutions found in each restriction scenario (A–E). Each point corresponds to a solution. Solutions were mapped into a 2-dimensional plot by Principal Coordinate Analysis of a distance matrix computed from the similarity between the density of all pairs of solutions (see text for details). Since original distances are preserved, scale was kept constant across plots to allow direct comparison. Colors refer to the values taken by the maximum variance of the Gamma distribution used to assign management intervals to landowners (see text for details). Higher values mean higher uncertainty assumed in the simulations as to the timing of management operations. (TIF) [file pone.0086001.s010.tif]
