# Supplementary material for: Optimization of Landscape Services under Uncoordinated Management by Multiple Landowners
Source: PLoS One. 2014 Jan 17;9(1):e86001. doi: 10.1371/journal.pone.0086001 (PMC3895036; doi:10.1371/journal.pone.0086001)
Supplement: Source Code S1 — Source code (R and C languages) of the simulation-optimization algorithms. All the code for the simulation-optimization algorithms was written within the original source code of the “mco” R package [56], for performance reasons. (ZIP) [file pone.0086001.s011.zip › Porto_LandscapeOptimization_source_code_and_results/README.htm]

Using the simulation-optimization script


# USING THIS SCRIPT

Requires R software.  
Re-running optimizations requires a Linux operating system.

## Basic usage

1. Start R.
2. Set working directory to the folder where you unpacked all the files: menu File | Change dir... or type `setwd('full-path-to-unpacked-files')`?
3. Load "run-optimizations.r": menu File | Source R code... or type `source('run-optimizations.r')`

### To produce the plots presented in the article (and others not presented)

*all plots are saved as TIFF files under the directory set above*

1. Choose option 1: "Explore results from paper".
2. Choose one of the runs to plot. The main run that was thoroughly analysed in the paper is `optim_020_100_100_1_400g.rdata`. The other runs explore the influence on *a priori* assumptions on results (see below).
3. Choose the restriction scenario
4. Choose the type of plot

### To examine the influence of *a priori* assumptions on solutions

1. Choose option 4: "Influence of a priori assumptions"
2. Choose option 4: "Compare solutions across different optimizations (signatures and ordination)"
3. Choose one of the runs:

- `optim_020_100_100_--_200g.rdata`: influence of varying mean-variance correlation of uncertainty
- `optim_020_100_---_1_400g.rdata`: influence of varying max uncertainty in implementation
- `optim_---_---_100_1_400g.rdata`: influence of varying mean landholding area of base landscapes

## To run other optimizations

*this requires a Linux operating system*

All the code to conduct simulation-optimization is written within the original `mco` package, in a modified version. You must compile the modified version and install it, in order to run optimizations.

### Compiling the C code

1. Open a terminal
2. Type the commands:

- `cd 'full-path-to-unpacked-files'`
- `R CMD REMOVE mcomp`
- `R CMD build mcomp`
- `R CMD INSTALL mcomp_1.0.12.tar.gz`

NOTE: you must ensure you have write permissions in the R library directory. If any of the above fails, precede the commands with `sudo`, to run as root.

### Making random base landscapes

*you must create random landscapes (only once) prior to run any optimization*

1. Run the steps in Basic usage above
2. Choose option 2: "Make random landscapes"
3. Enter number of landscapes to produce
4. Enter mean property area (main optimization needs a value of 20 here)
5. Enter variance of property area (main optimization needs a value of 100 here)

An excerpt of each landscape is printed on the screen, for progress and debugging purposes. Landscapes are saved in a .bin file (which may be quite huge).

### Running optimizations

*any of these will take days to complete*

1. Run the steps in Basic usage above
2. Choose option 3 (main optimization) or 4 (influence of *a priori* assumptions).
3. If choosing 4, then choose an option from 1 to 3. Note that you have to produce the base landscapes needed to run any of these; you'll be warned of missing files.

Optimization results are saved in an .rdata file. You must copy it to Results folder to explore the results.

### Examining solutions in detail

*this consumes huge amounts of memory*

1. Run the steps in Basic usage above
2. Choose option 5: "Simulate solutions along time"
3. Choose one or more solutions to simulate, or type a solution manually. Do not run for more than 3 solutions simultaneously, due to high memory consumption.

## Customization

This script, used as provided, is expected to reproduce the results in the article apart from minor variations due the stochastic nature. However, several options can be changed rather easily by editing "run-optimizations.r".

### Optimization objectives

6 objectives were used in the article. The first 4 are fixed, the remaining 2 (biodiversity objectives, i.e. minimum area of given age classes) can be changed or even increased. For that, edit the following lines:

maxInterval=100  
...  
otherpars=list(  
   pop=800L  
   ,gener=as.integer(c(10, 20, 50, 100, 150, 200, 250, 300, 350, 400))  
   **,intervals=as.integer(c(30,60,9999))  
   ,minareaTSD=c(0.10,0.10)**  
   ,minInterval=3  
   ,maxInterval=as.integer(maxInterval)  
   ,n.years=100L  
   ...  
)

Where `intervals` are the limits of age classes (30-60 and >60 years in this case) and `minareaTSD` the minimum area proportion (relative to the whole landscape) that must be guaranteed during the whole simulation period (10% of each class in this case). You can add more classes and corresponding proportions, each will correspond to an objective. Note that the length of `minareaTSD` is equal to the length of `intervals` minus 1. Example:

,intervals=as.integer(c(10,20,30,50))  
,minareaTSD=c(0.20,0.10,0.15)

### Simulation and optimization options

Changing how many years the landscape simulator runs:

,n.years=100L

NOTE: you must append L to the number to specify that it is an integer

Changing the population and number of generations of the optimization algorithm:

,pop=800L

Changing the minimum and maximum values that the prescribed management interval can take:

maxInterval=100  
,minInterval=3

(note that the management interval is just the mean of a gamma random number generation)

## Files contained

### Main directory

- **README.htm** - This file.
- **run-optimizations.r** - Main script to either load optimization results from the article, or run new optimizations START HERE
- **plotting\_functions.r** - Functions for plotting and visualizing optimization and simulation results, used internally
- **helper\_functions.r** - Accesory functions used internally
- **mcomp (directory)** - Modified version of `mco` package, which includes all the code for landscape generator/simulator/analyzer and the objective function used in optimization.
- **Results (directory)** - Optimization results analyzed in the paper

### /Results directory

- **optim\_020\_100\_100\_1\_400g.rdata** - Results of the main optimization run
- **optim\_---\_---\_100\_1\_400g.rdata** - Results exploring the influence of changing landscape properties
- **optim\_020\_100\_---\_1\_400g.rdata** - Results exploring the influence of changing implementation uncertainty
- **optim\_020\_100\_100\_--\_200g.rdata** - Results exploring the influence of changing the correlation between mean and variance of implementation uncertainty (200 generations only)

### /mcomp directory

Many files of the original `mco` package, plus new source files for the landscape generator/simulator/analyzer
